# The Microbiome and Metabolites in Fermented Pu-erh Tea as Revealed by High-Throughput Sequencing and Quantitative Multiplex Metabolite Analysis

**DOI:** 10.1371/journal.pone.0157847

**Published:** 2016-06-23

**Authors:** Yongjie Zhang, Ida Skaar, Michael Sulyok, Xingzhong Liu, Mingyong Rao, John W. Taylor

**Affiliations:** 1 School of Life Sciences, Shanxi University, Taiyuan, Shanxi, China; 2 Department of Plant and Microbial Biology, University of California, Berkeley, California, United States of America; 3 Section of Mycology, Norwegian Veterinary Institute, Oslo, Norway; 4 Center for Analytical Chemistry, Department of Agrobiotechnology, University of Natural Resources and Life Sciences, Tulln, Austria; 5 State Key Laboratory of Mycology, Institute of Microbiology, Chinese Academy of Sciences, Beijing, China; 6 Bureau of Culture and Sports, Puer, Yunnan, China; Oklahoma State University, UNITED STATES

## Abstract

Pu-erh is a tea produced in Yunnan, China by microbial fermentation of fresh *Camellia sinensis* leaves by two processes, the traditional raw fermentation and the faster, ripened fermentation. We characterized fungal and bacterial communities in leaves and both Pu-erhs by high-throughput, rDNA-amplicon sequencing and we characterized the profile of bioactive extrolite mycotoxins in Pu-erh teas by quantitative liquid chromatography-tandem mass spectrometry. We identified 390 fungal and 629 bacterial OTUs from leaves and both Pu-erhs. Major findings are: 1) fungal diversity drops and bacterial diversity rises due to raw or ripened fermentation, 2) fungal and bacterial community composition changes significantly between fresh leaves and both raw and ripened Pu-erh, 3) aging causes significant changes in the microbial community of raw, but not ripened, Pu-erh, and, 4) ripened and well-aged raw Pu-erh have similar microbial communities that are distinct from those of young, raw Ph-erh tea. Twenty-five toxic metabolites, mainly of fungal origin, were detected, with patulin and asperglaucide dominating and at levels supporting the Chinese custom of discarding the first preparation of Pu-erh and using the wet tea to then brew a pot for consumption.

## Introduction

Tea is one of the most popular and widely consumed beverages in the world. It is normally produced from the leaves of two varieties of the tea plant, *Camellia sinensis* var. *sinensis* and var. *assamica* [1]. Tea has important physiological effects on consumers due to the presence of compounds such as polyphenols, amino acids, vitamins, carbohydrates, caffeine, and purine alkaloids, all of which can have health benefits [2–4]. Among the claimed effects of consuming tea are blood lipid and weight reduction, antimicrobial, antioxidant, and anticancerogenic activities, and enhanced digestion [4, 5]. Based on processing procedures, tea can be divided into at least six different types: green, yellow, white, oolong, black (called red tea in China), and post-fermented tea (called dark tea in China) [6]. Of them, post-fermented tea is unique due to the microbial fermentation process, which may last from several months to many years. Post-fermented Chinese teas include Fu-zhuan in Hunan, Qing-zhuan in Hubei, Liu-bao in Guangxi, and Pu-erh in Yunnan, the latter being best known, both for its taste and its political economics [7].

Pu-erh tea has been made from *C*. *sinensis* var. *assamica* since the Tang dynasty (AD 618–906). There are two types: naturally fermented (raw) and purposely fermented (ripened) (Figure A in S1 File). For raw Pu-erh, the initial processing starts with natural withering of fresh tea leaves to initiate their drying, roasting of leaves to continue drying and denature plant enzymes, rolling the leaves to remove additional moisture, and, finally, complete the drying process through direct exposure to the sun [8]. The dry, raw Pu-erh is then aged for varying periods to promote natural, solid substrate fermentation. To some extent, the quality and flavor of raw Pu-erh tea improves with age [9], and consequently aged raw Pu-erh is more valuable. Ripened Pu-erh, which was developed in the 1970s in order to shorten the aging process needed for raw Pu-erh, is produced in the same way as raw Pu-erh but with an additional step called “pile fermentation”, a microbial fermentation of the tea initiated by the addition of water [1, 10, 11]. Finished Pu-erh tea, both raw and ripened, can be left as loose leaves or compressed into cakes or bricks to facilitate transport and storage.

The production and quality of Pu-erh is closely related to microbial activity [12], making it important to understand the Pu-erh microbiome. Previous studies have investigated fungi and/or bacteria emerging during the pile fermentation of ripened Pu-erh, using either: 1) culture-dependent [13–15], 2) first-generation, culture-independent approaches such as denatured gradient gel electrophoresis [15–17] and Sanger, clone library sequencing [18] as well as, 3) metagenomic sequencing [19, 20]. *Aspergillus niger* and *Blastobotrys adeninivorans* were frequently documented as dominant lineages in Pu-erh from both culture-dependent and culture-independent studies. Some other studies have investigated the microbial diversity in Pu-erh teas of different ages [21, 22], but none have characterized the microbial communities of fresh leaves, raw Pu-erh, and ripened Pu-erh, nor attempted to correlate bacterial and fungal community composition with environmental factors (e.g, the type and age of tea, the tea producer).

As a product of microbial fermentation, the safety of Pu-erh tea is a topic of continued concern. Toxic microbial metabolites were investigated from Pu-erh tea samples or fungal isolates recovered from Pu-erh, but inconsistent results were found in literatures. Some studies detected no mycotoxins [15, 23, 24], but other studies detected mycotoxins such as aflatoxin B1, deoxynivalenol, and ochratoxin A [25–28]. Previous studies, however, have not related microbial community composition to the production of potentially toxic microbial metabolites.

Recent advances in massively parallel, short-amplicon, sequencing technologies have launched a breakthrough in microbial ecology studies of the fermentation of wine, milk, and other foods [29–37]. We employed high-throughput amplicon sequencing to investigate the microbiome in fresh tea leaves, raw and ripened Pu-erh, and then performed multiplex analysis of metabolites in the tea samples. Our goals were 1) to identify microbial diversity and composition in Pu-erh; 2) to compare microbial community structure among fresh tea leaves, and raw and ripened Pu-erh tea; 3) to identify potential factors affecting microbial communities in Pu-erh tea, and 4) to identify microbial metabolites in Pu-erh tea.

## Materials and Methods

### Samples used in this study

Seven samples of fresh leaves of *Camellia sinensis* var. *assamica* and 31 Pu-erh tea samples were examined (Table A in S1 File). Fresh leaves were collected in three different tea gardens located in Pu-erh City of southern Yunnan Province, southwest China. The 31 tea samples, 15 raw and 16 ripened, were collected from five different companies in Pu-erh City and had been stored for 0–28 years (raw) and 0–13 years (ripened) (Table A in S1 File). They were either loose or compressed as cakes or bricks. All samples were subject for high throughput sequencing. The 31 tea samples were also analyzed for fungal and bacterial bioactive extrolites.

### DNA extraction, amplification, and Sanger sequencing of plant gene fragments

DNA suitable for PCR amplification of plant, fungal and bacterial gene fragments was obtained using the MoBio PowerSoil DNA Isolation Kit (MoBio Laboratories, Carlsbad, CA, USA), which involves mechanical lysis, chemical lysis, and DNA purification.

To check the botanical identity of the teas, we amplified two plastid DNA markers of plants: the maturase K gene (*matK*) and the large subunit of the ribulose 1, 5-bisphosphate carboxylase ⁄oxygenase gene (*rbcL*). We used primers matK472F and matK1248R for *matK* [38], and rbcLa_F and rbcLa_R for *rbcL* [39]. PCR was carried out in 25 μl reactions containing 2.5 μl 10 × PCR Buffer, 2.5 μl 2 mM dNTPs, 1 μl each 10 μM primer, 5 μl 5 × Q-Solution, 1.5 μl 100 μg/μl BSA (bovine serum albumin), 1 μl 25 mM MgCl_2_, 0.2 μl HotStar Taq Plus DNA Polymerase (Qiagen, Valencia, CA, USA), and 1 μl DNA template. PCR conditions were: denaturation at 95°C for 5 min, 40 amplification cycles of 30 sec at 94°C, 30 sec at 48°C (for *matK*) or 52°C(for *rbcL*), and 1 min at 72°C; followed by a 10 min final extension at 72°C. PCR amplicons were purified with ExoSAP-IT reagent (USB, Cleveland, OH) following manufacturer's instructions and then sequenced on an ABI 3730 XL 96-capillary array DNA analyzer using Life Technologies BigDye terminator version 3.1 at the UC Berkeley DNA Sequencing Facility. Chromatogram files were viewed using FinchTV 1.4.0 (http://www.geospiza.com/Products/finchtv.shtml). Sequences were aligned using Muscle 3.8 [40].

#### Amplification and Illumina Miseq sequencing of fungal ITS and bacterial SSU fragments

From the genomic DNA solutions, we amplified both the nrDNA ITS1 region of fungi using primers ITS1F and ITS2 [41, 42] and the V4 hypervariable region of the 16S rRNA gene of bacteria using primers 515f and 806r [43]. A 12-nt barcode unique for each sample was included in reverse primers. PCR was carried out in the same reactions as plant fragment amplification but without the Q-Solution. PCR conditions were: denaturation at 95°C for 5 min, 35 amplification cycles of 30 sec at 94°C, 30 sec at 50°C, and 1 min at 72°C; followed by a 10 min final extension at 72°C. Samples were PCR-amplified in triplicate, and the triple amplicons of each sample were pooled before cleaning using the Agencourt AMPure XP PCR purification kit (Beckman Coulter Genomics, Danvers, MA, USA).

Purified amplicons were individually quantified using the Qubit dsDNA HS assay kit (Invitrogen, Eugene, Oregon, USA) on the Qubit flourometer (Invitrogen, Carlsbad, CA, USA) and pooled in equimolar concentrations into two composite samples (one for fungi and one for bacteria). Concentrations of the pooled amplicons and length distribution were measured in an Agilent 2100 Bioanalyzer at the Functional Genomics Laboratory of UC Berkeley. Fungal amplicons and bacterial amplicons were pooled at a 2:1 ratio and sent to the Stanford University Functional Genomics Facility for 250 bp paired-end sequencing on an Illumina Miseq platform. Fungal and bacterial sequencing primers were also pooled for each read before submission to the sequencing facility.

### Bioinformatics of high-throughput data

Sequence de-multiplexing and bioinformatic processing were performed with the QIIME 1.8.0 [44] and the UPARSE [45] pipelines. Forward and reverse raw reads from the sequencing facility were first trimmed with CutAdapt1.4.2 [46] to the point where the sequence met the distal priming site, and further trimmed using Trimmomatic 0.32 [47] to remove any additional low quality end regions. Reads were paired using USEARCH v 7.0.1090 with a minimum Phred score sequence cutoff threshold of 3 and a minimum sequence length of 75 bp. After discarding those reads with > 0.5 expected errors, paired reads were de-multiplexed into a fungal dataset and a bacterial dataset.

For each dataset, identical sequences were de-replicated, and singleton sequences were discarded. Remaining sequences were grouped into operational taxonomic units (OTUs) in USEARCH with 97% similarity cutoff. Reference-based chimera filtering was performed against the UNITE database [48] for the fungal dataset or against the “Gold” database (http://sourceforge.net/projects/microbiomeutil/files/) for the bacterial dataset. OTUs were classified taxonomically using a QIIME-based wrapper of BLAST against the UNITE database [49] for fungi or against the Greengenes database [50] for bacteria. To discard non-target sequences, unassigned fungal sequences were further evaluated by ITSx 1.0.9 [51], and unassigned bacterial OTU sequences were further evaluated by Metaxa 1.1.2 [52]. Use of these tools would exclude from the fungal data set erroneously assigned bacterial SSU sequences and fungal mitochondrial SSU sequences, and exclude from the bacterial data set erroneously assigned fungal ITS sequences, archaeal/eukaryotic nuclear SSU sequences and chloroplast or mitochondrial DNA sequences. Bacterial OTU representative sequences were aligned using Muscle [40], and, after filtering the top 10% most entropic base positions, a phylogenetic tree was constructed using FastTree [53]. Representative sequences of fungal OTUs were deposited in GenBank under accession numbers KT359915-KT360304 and bacterial OTUs under KT360305-KT360933.

OTU tables were constructed by mapping reads to OTUs (−usearch_global -strand plus -id 0.97) and by applying the python script uc2otutab.py (http://drive5.com/python/). Any OTUs representing less than 0.005% of the total sequences in the OTU table were removed to avoid inclusion of erroneous reads that would inflate estimates of diversity [54]. To compare samples on an equal basis, all samples were rarefied to even sampling depths prior to statistical analysis. Rarefaction depths were set to maximize the number of samples included while still maintaining a reasonable number of sequences. Specifically, when comparing among fresh leaf, raw and ripened Pu-erh samples, we rarefied the fungal dataset to 39 507 sequences per sample by keeping all samples; we rarefied the bacterial dataset to 1 103 sequences per sample by discarding three fresh leaf samples (LN2, LS2, and LS4) and three raw Pu-erh samples (A3, A14, and A15). When focusing just on raw Pu-erh samples, we rarefied the fungal dataset to 60 926 sequences per sample and the bacterial dataset to 1 268 sequences per sample, after removing from the dataset three raw Pu-erh samples (A3, A14, and A15). When focusing just on ripened Pu-erh samples, we rarefied the fungal dataset to 94 539 sequences per sample and the bacterial dataset to 16 512 sequences per sample.

### Statistical analysis of microbial community

Statistical analyses relied on QIIME [44] and R [55]. We calculated a number of common metrics used in community ecology, including α-diversity (observed richness, Chao 1, Shannon, and Simpson Evenness) and β-diversity (Bray-Curtis, Binary-Jaccard, or weighted-UniFrac). Principal coordinates were computed from the resulting β-diversity distance matrices to compress multiple dimensions into three dimensional principal coordinate analysis (PCoA) plots, enabling visualization of microbial community relationships. A nonmetric multidimensional scaling (NMDS) ordination of the resulting β-diversity distance matrices among samples was also carried out to summarize patterns of fungal/bacterial community structures. ANOSIM and permutational MANOVA (ADONIS) with 999 permutations were used to test significant differences between sample groups based on β-diversity distance matrices.

To compare the whole community overlap between fresh leaf, raw and ripened Pu-erh, we generated a Venn diagram to illustrate the proportion of shared and unique taxa using the 3 Way Venn Diagram Generator (http://jura.wi.mit.edu/bioc/tools/venn3way/index.php). To identify the specific OTUs that characterize fresh leaf, raw Pu-erh and ripened Pu-erh, we used the ‘indicspecies’ package [56] in R. We also tested for potential correlation between fungal and bacterial community composition using a Mantel test based on Binary-Jaccard or Bray-Curtis distance matrices.

### Multiplex analysis of fungal and bacterial metabolites

Milled tea samples were weighed into 50-ml polypropylene tubes, and the extraction solvent (acetonitrile/water/acetic acid 79:20:1, v/v/v) was added at a ratio of 5 ml of solvent per gram of sample. Samples were extracted for 90 min on a GFL 3017 rotary shaker (GFL, Burgwedel, Germany), diluted with the same volume of extraction solvent, and the diluted extracts injected [57]. Centrifugation was not necessary due to sufficient sedimentation by gravity. Apparent recoveries of the analytes were determined by spiking five different samples with a multi-analyte standard on one concentration level. The spiked samples were stored overnight at ambient temperature to allow evaporation of the solvent and to establish equilibrium between the analytes and the sample. The extraction, dilution and analysis were as described previously [57].

The chromatographic method and the chromatographic and mass spectrometric parameters are as described by Malacova *et al*. [58]. Briefly, LC-MS/MS screening of target microbial metabolites was performed with a QTrap 5500 LC-MS/MS System (Applied Biosystems, Foster City, CA, USA) equipped with TurboIonSpray electrospray ionization (ESI) source and a 1290 Series HPLC System (Agilent, Waldbronn, Germany). Chromatographic separation was performed at 25°C on a Gemini^®^ C18-column, 150 × 4.6 mm i.d., 5 μm particle size, equipped with a C18 4 × 3 mm i.d. security guard cartridge (Phenomenex, Torrance, CA, USA). ESI-MS/MS was performed in the time-scheduled multiple reaction monitoring (MRM) mode both in positive and negative polarities in two separate chromatographic runs per sample by scanning two fragmentation reactions per analyte. The MRM detection window of each analyte was set to its expected retention time ±27 s and ±48 s in the positive and the negative modes, respectively. Confirmation of positive analyte identification was obtained by the acquisition of two MRMs per analyte (with the exception of moniliformin which exhibited only one fragment ion). This approach yielded 4.0 identification points according to European Union Commission decision 2002/657 (EU2002). In addition, the LC retention time and the intensity ratio of the two MRM transitions agreed with the related values of an authentic standard within 0.1 min and 30% rel., respectively.

## Results

### Plant DNA fragment analyses in fresh leaves and Pu-erh tea samples

Direct sequencing of *rbcL* amplicons was used to test for *C*. *sinensis* sequences in all samples of leaves and tea because it amplified more reliably than *matK*. Chromatograms of *rbcL* Sanger sequences showed homozygous nucleotide peaks in all but four ripened Pu-erh samples (B4, B8, B9, and B11) (Figure B in S1 File). For these samples, we visually inspected the heterozygous chromatogram peaks and found nucleotides characteristic of *C*. *sinensis* at the variable positions, indicating the coexistence of *C*. *sinensis* and other plants in these samples. Three other ripened Pu-erh samples (B2, B7, and B13) yielded homozygous DNA sequences that, in subsequent BLAST searches, represented species of other plant genera, i.e, *Musa*, *Pinus*, and *Brassica*. Microscopic examination and tasting of tea made from these three samples did not find obvious differences between them and other ripened Pu-erh samples. To check for the presence of *C*. *sinensis* in these samples, five individual leaves were selected from each sample and independently processed to yield *rbcL* sequence. From these samples, which provided homozygous and heterozygous chromatograms of *C*. *sinensis* and the other plants, *C*. *sinensis rbcL* sequences could be observed in each sample. No variation in *C*. *sinensis rbcL* sequences could be detected among any of the samples.

### Microbial taxon richness and community composition

We successfully amplified and sequenced DNA from fungal and bacterial communities from all samples. After removing primers and low-quality ends, merging paired reads and de-multiplexing, each sample provided more than 42 383 fungal ITS sequences (7 587 458 in total) and 29 721 bacterial SSU sequences (6 949 251 in total). Further processing to remove singleton sequences, chimeras, non-target sequences and low-abundance OTUs reduced the yield to between 39 507 and 214 960 fungal sequences per sample (7 305 834 total) and 66 to 305 472 bacterial sequences per sample (2 413 213 total). The low number of bacterial sequences detected in fresh leaf and some raw Pu-erh samples was due to amplification of competing chloroplast SSU DNA, which accounted for 86.23% in fresh leaf samples and 65.81% in raw Pu-erh samples, compared to just 0.09% in ripened Pu-erh samples (Figure C in S1 File). These chloroplast sequences were excluded prior to further analysis. The final number of OTUs passing abundance filtering (at 0.005%) was 390 for fungi (65 to 175 OTUs per sample) and 629 for bacteria (18 to 466 OTUs per sample with the lower numbers found in fresh leaves and raw Pu-erh). The most commonly observed fungal taxa belonged to Ascomycota (305 OTUs; 91.67% of total sequences); the most commonly observed bacterial taxa belong to Firmicutes (220 OTUs; 37.01% of total sequences), Actinobacteria (172 OTUs; 43.16%), and Proteobacteria (158 OTUs; 13.89%) (Figure D in S1 File).

### Comparison of microbial community in fresh leaves, raw and ripened Pu-erh

**Fig 1:**
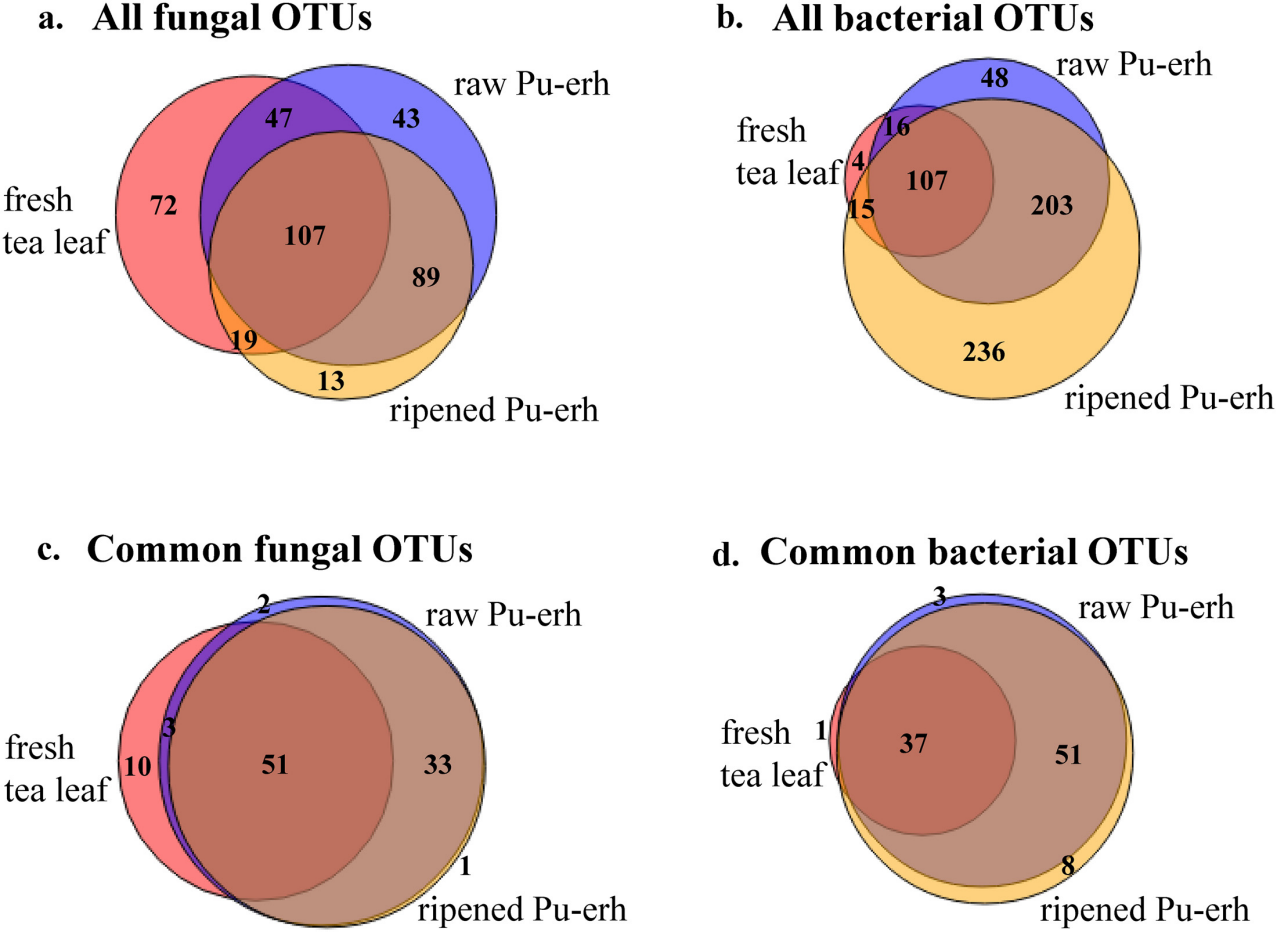
OTU overlap among fresh tea leaves (red), raw (blue) and ripened (orange) Pu-erh tea samples. Venn diagrams illustrate the number of unique and shared fungal (a, c) and bacterial (b, d) OTUs. We compared both the total OTUs (a, b) and just the first 100 most abundant OTUs (c, d) in the fungal/bacterial datasets.

Using all sequences, about 1/4 of the fungal OTUs (107/390) and 1/6 of the bacterial OTUs (107/629) were shared among the three sample types (Fig 1a and 1b), indicative of significant variation in community composition among sample types. When comparing OTUs between fresh leaf and Pu-erh tea (raw and ripened), 54% of fungal OTUs (173/318) or 22% of bacterial OTUs (138/625) present in Pu-erh were also found in fresh leaf samples (Fig 1a and 1b), suggesting that fresh leaves are an important microbial reservoir for Pu-erh fermentation. When comparing raw and ripened Pu-erh teas, 62% of fungal OTUs (196/318) and 50% of bacterial OTUs (310/625) were shared. To evaluate the effect on microbial community similarity of the many rare OTUs found in each sample type, we analyzed OTUs shared among sample types using just the 100 most abundant fungi and bacteria. Focusing on these most abundant microbes, the fraction of shared fungal and bacterial OTUs rose dramatically (e.g, from 17–27% to 37–51% for shared OTUs by three types), irrespective of whether all three types or any two types were compared (Fig 1c and 1d).

**Fig 2:**
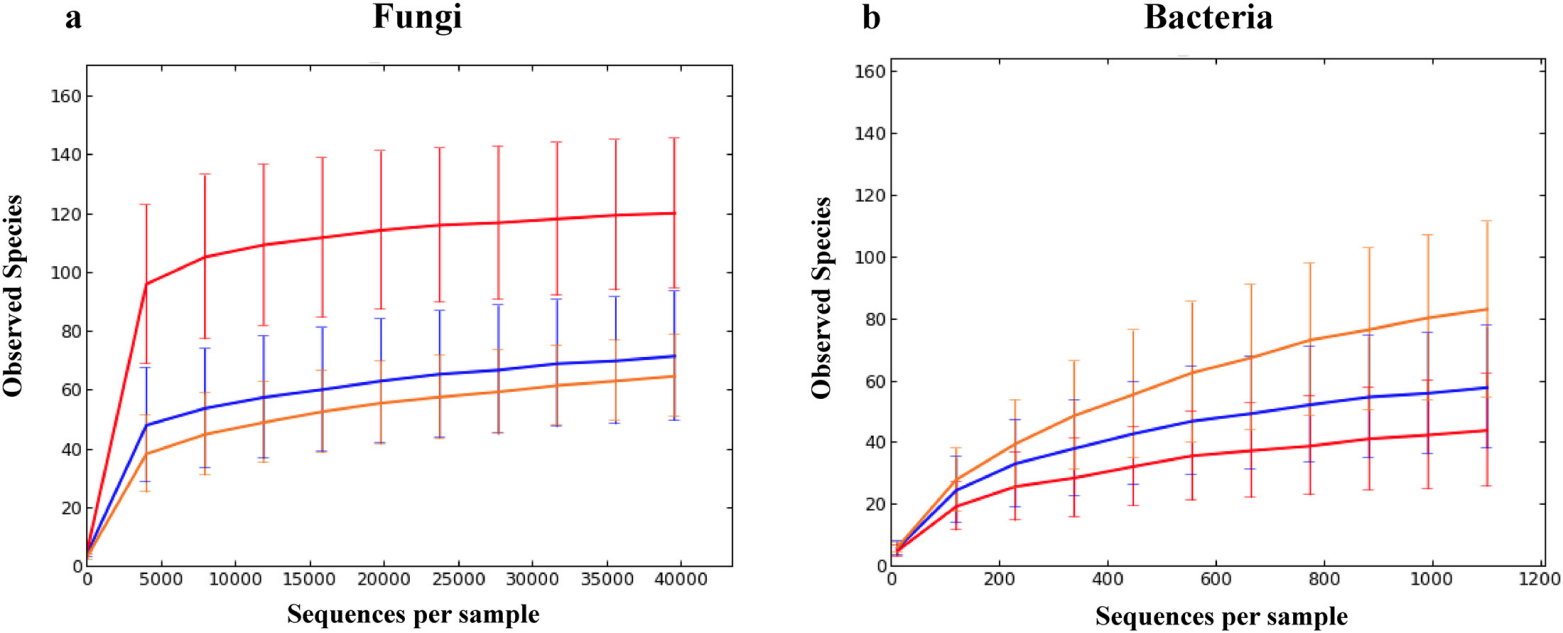
Rarefaction-based comparison of fresh tea leaf (red), raw (blue) and ripened (orange) Pu-erh samples with regard to fungal (a) and bacterial (b) richness. Fungi were rarefied at 39 507 sequences to keep all samples, while bacteria at 1103 sequences to exclude three fresh leaf samples (LN2, LS2, and LS4) and three raw Pu-erh samples (A3, A14, and A15).

Reexamining α-diversity after rarefying to the same sequencing depth, fresh leaves had more fungal OTUs but fewer bacterial OTUs than Pu-erh tea (Fig 2). For fungi, the difference between fresh leaves and Pu-erh was significant in α-diversity indices (observed species, Chao1, Shannon, and Simpson-e), while the difference between raw and ripened Pu-erh was not significant (Figure E a-d in S1 File). For bacteria, ripened Pu-erh showed significantly higher richness than either fresh leaves or raw Pu-erh with the observed species or Chao1 estimates, but not with Shannon or Simpson-e indices (Figure E e-h in S1 File).

**Fig 3:**
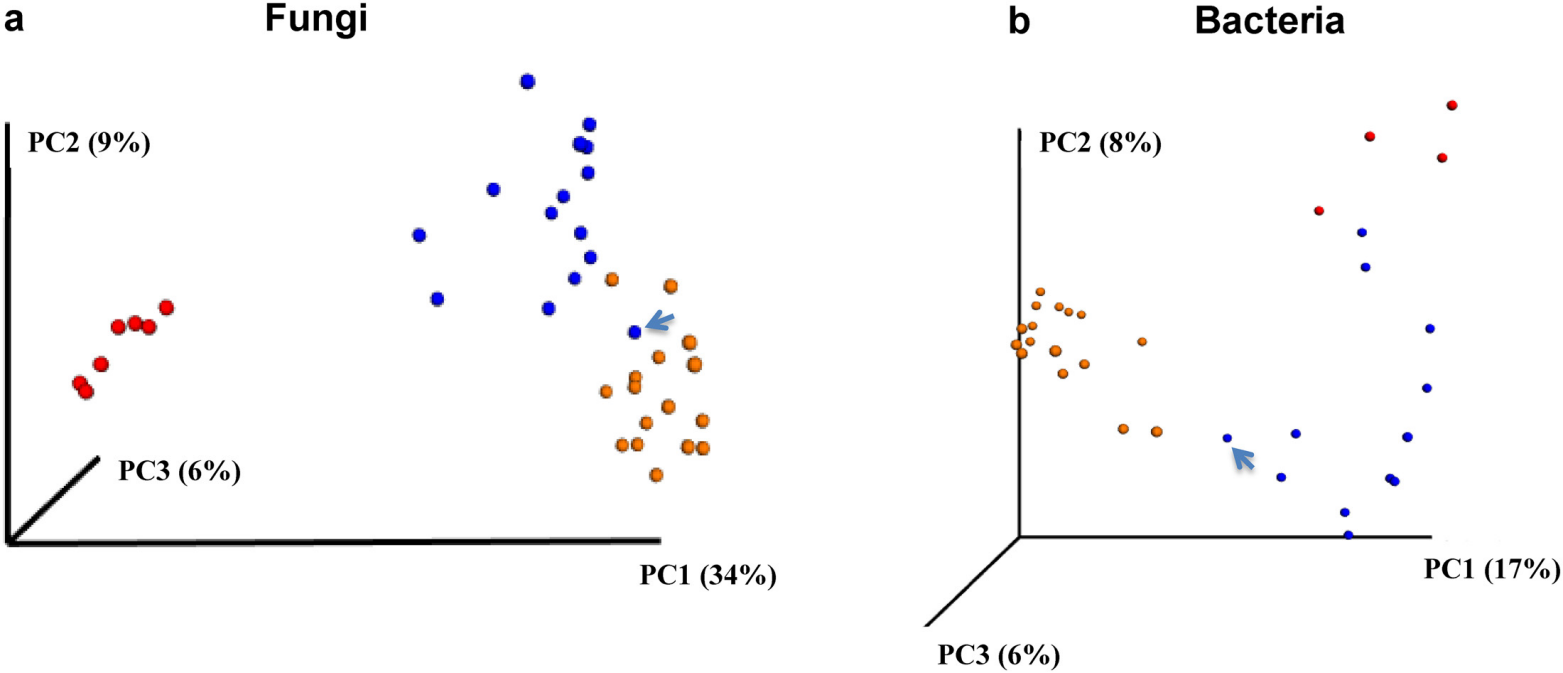
PCoA of Binary-Jaccard dissimilarities of microbial communities of fresh tea leaf (red), raw (blue) and ripened (orange) Pu-erh samples. The oldest raw Pu-erh sample (A6, 28 years old), indicated by an arrow in both PCoA analyses, is more similar to ripened Pu-erh than to other raw Pu-erh samples.

Considering β-diversity, the microbial community differences among fresh leaves, raw and ripened Pu-erh samples were highly significant in both ANOSIM and ADONIS tests using Binary-Jaccard and Bray-Curtis β-diversity estimates (Table 1). The type of sample, leaf, raw or ripened Pu-erh, explained 20–40% of total community difference. Similar results were also visualized in PCoA (Fig 3) and NMDS plots (Figure F in S1 File). Most interestingly, the oldest Pu-erh sample (A6) tended to cluster with ripened Pu-erh samples, especially when considering the fungal community (Fig 3a).

**Table 1:** Comparison among fresh leaf, raw Pu-erh and ripened Pu-erh samples at β-diversity level.

	Fungi		Bacteria		
	Binary-Jaccard	Bray-Curtis	Binary-Jaccard	Bray-Curtis	weighted-Unifrac
All data					
ANOSIM					
R	0.683	0.685	0.802	0.687	0.572
*P*	0.001	0.001	0.001	0.001	0.001
ADONIS					
R^2^	0.391	0.338	0.209	0.234	0.290
*P*	0.001	0.001	0.001	0.001	0.001
Fresh leaf vs. raw Pu-erh					
ANOSIM					
R	0.999	0.761	0.422	0.392	0.480
*P*	0.001	0.001	0.007	0.010	0.002
ADONIS					
R^2^	0.376	0.253	0.132	0.151	0.203
*P*	0.001	0.001	0.001	0.004	0.005
Fresh leaf vs. ripened Pu-erh					
ANOSIM					
R	1.000	1.000	0.944	0.985	0.956
*P*	0.001	0.001	0.001	0.002	0.001
ADONIS					
R^2^	0.503	0.424	0.181	0.195	0.360
*P*	0.001	0.001	0.001	0.001	0.001
Raw Pu-erh vs. ripened Pu-erh					
ANOSIM					
R	0.376	0.451	0.818	0.622	0.442
*P*	0.001	0.001	0.001	0.001	0.001
ADONIS					
R^2^	0.134	0.195	0.155	0.179	0.166
*P*	0.001	0.001	0.001	0.001	0.001

Many indicator taxa were found for fresh leaf, raw and ripened Pu-erh samples (Table 2). As expected, lineages of thermophilic or thermotolerant fungi (e.g, *Rhizomucor pusillus* and *Thermomyces lanuginosus*) and bacteria (e.g, *Bacillus coagulans*, *Bacillus thermoamylovorans*, and *Tuberibacillus calidus*) were among the indicator taxa found in ripened Pu-erh samples (Table B in S1 File). Two of the indicator taxa found for Pu-erh (raw and ripened), *Aspergillus niger* and *Blastobotrys adeninivorans*, have been considered to be dominant fungal lineages in Pu-erh from both culture-dependent and culture-independent studies [15, 16].

**Table 2:** Number of indicator fungal/bacterial OTUs detected for fresh leaf, raw and ripened Pu-erh samples.

	Fresh leaf	Raw Pu-erh	Ripened Pu-erh	Raw+Ripened Pu-erh
Fungi				
No. total OTUs	245	286	228	318
No. indicator OTUs	135	10	20	40
Bacteria				
No. total OTUs	142	374	561	625
No. indicator OTUs	22	18	307	12

### Effect of variables on microbial community difference in Pu-erh

We investigated four variables (age, producer, pure tea *vs*. tea contaminated with other plants, and loose *vs*. pressed tea) in addition to sample type to see if they could help explain the microbial community difference found between raw and ripened Pu-erh. To investigate the effect of aging, we binned our raw and ripened Pu-erh samples in either two (young and old) or three (young, middle aged, and old) age stages (Table A in S1 File). Among these variables, only age of tea showed a statistically significant effect with at least two methods of estimating β-diversity and the two methods of comparison (ANOSIM and ADONIS), and only for raw Pu-erh (Table C in S1 File).

A Mantel test was used to examine the correlation between fungal and bacterial communities. We found no correlation between communites of the two types of microbes in raw Pu-erh based on either Binary-Jaccard or Bray-Curtis distance matrices (Table D in S1 File). We found a significant correlation for the two types of communities in ripened Pu-erh using the Bray-Curtis distance matrices with all 16 samples (r = 0.410, *P* = 0.004), but not using Binary-Jaccard distance matrices or when excluding the seven ripened Pu-erh samples contaminated with different plant species (Table D in S1 File).

### Mycotoxigenic fungi and mycotoxins in Pu-erh teas

Reasoning that common and abundant fungi are more likely to pose a possible mycotoxin problem to consumers, we further reduced the number of fungi to a set of the 15 most abundant OTUs in a sample type, i.e, leaves, raw or ripened Pu-erh. These dominant fungal taxa were found in almost all such samples (Table 3), where they accounted for between 68% and 95% of the total sequences. Several of the fungi found in our Pu-erh samples are known mycotoxin producers, such as *Aspergillus niger* [59], *Aspergillus restrictus* [60], and *Penicillium citrinum* [61]. It is possible that some bacteria in Pu-erh produce toxins, but bacterial toxins are not addressed in this study. Fortunately, some frequently documented toxin-producing bacterial genera, such as *Clostridium*, *Escherichia*, *Vibrio*, and *Salmonella* [62], were not detected in our Pu-erh samples (Table E in S1 File).

**Table 3:** The first 15 most abundant fungal OTUs in fresh leaf, raw and ripened Pu-erh samples.

Abundance Rank	Fresh leaf			Raw Pu-erh			Ripened Pu-erh		
	OTU ID	Fungal taxon	OC ^a^	OTU ID	Fungal taxon	OC^a^	OTU ID	Fungal taxon	OC^a^
1	OTU_5 ^b^	*Cladosporium* sp.	7	OTU_2 ^b^	*Aspergillus* sp.	15	OTU_1	*Blastobotrys adeninivorans*	16
2	OTU_15	*Lophodermium* sp.	7	OTU_8	*Debaryomyces hansenii*	13	OTU_1310	*Aspergillus niger*	16
3	OTU_23	*Epicoccum nigrum*	7	OTU_1	*Blastobotrys adeninivorans*	15	OTU_2 ^b^	*Aspergillus* sp.	16
4	OTU_29	Tremellomycetes sp.	6	OTU_125	*Aspergillus penicillioides*	15	OTU_1626	*Aspergillus penicillioides*	16
5	OTU_32	Ascomycota sp.	7	OTU_1626	*Aspergillus penicillioides*	15	OTU_4	*Thermomyces lanuginosus*	16
6	OTU_24	No blast hit	7	OTU_1199	*Aspergillus cibarius*	15	OTU_1895	Trichocomaceae sp.	16
7	OTU_78	Ascomycota sp.	6	OTU_9	Myriangiales sp.	15	OTU_6	*Penicillium citrinum*	16
8	OTU_68	*Colletotrichum xanthorrhoeae*	7	OTU_1936	*Aspergillus penicillioides*	15	OTU_13	*Aspergillus restrictus*	16
9	OTU_34	*Selenophoma mahoniae*	7	OTU_1825	*Aspergillus cibarius*	15	OTU_11	Trichocomaceae sp.	16
10	OTU_50	*Pestalotiopsis foedans*	7	OTU_5 ^b^	*Cladosporium* sp.	15	OTU_1554	*Aspergillus penicillioides*	16
11	OTU_1891	*Strelitziana africana*	7	OTU_17	*Neurospora terricola*	15	OTU_125	*Aspergillus penicillioides*	16
12	OTU_48	*Sporobolomyces oryzicola*	7	OTU_22	*Neurospora terricola*	14	OTU_1825	*Aspergillus cibarius*	16
13	OTU_25	*Septoria aegopodina*	7	OTU_13	*Aspergillus restrictus*	13	OTU_1936	*Aspergillus penicillioides*	16
14	OTU_238	*Peyronellaea sancta*	7	OTU_1249	*Aspergillus penicillioides*	15	OTU_1061	*Aspergillus subversicolor*	11
15	OTU_55	*Strelitziana mali*	5	OTU_1310	*Aspergillus niger*	15	OTU_30	*Penicillium brocae*	15

^a^ Occurrence (OC) indicates the detection of a given OTU in the seven fresh leaf samples, the 15 raw Pu-erh samples, or the 16 ripened Pu-erh samples.

^b^ The taxonomy of OTU_2 and OTU_5 were initially assigned as Eurotiales sp. and Fungi sp, respectively. They were revised according to online Blast search

**Fig 4:**
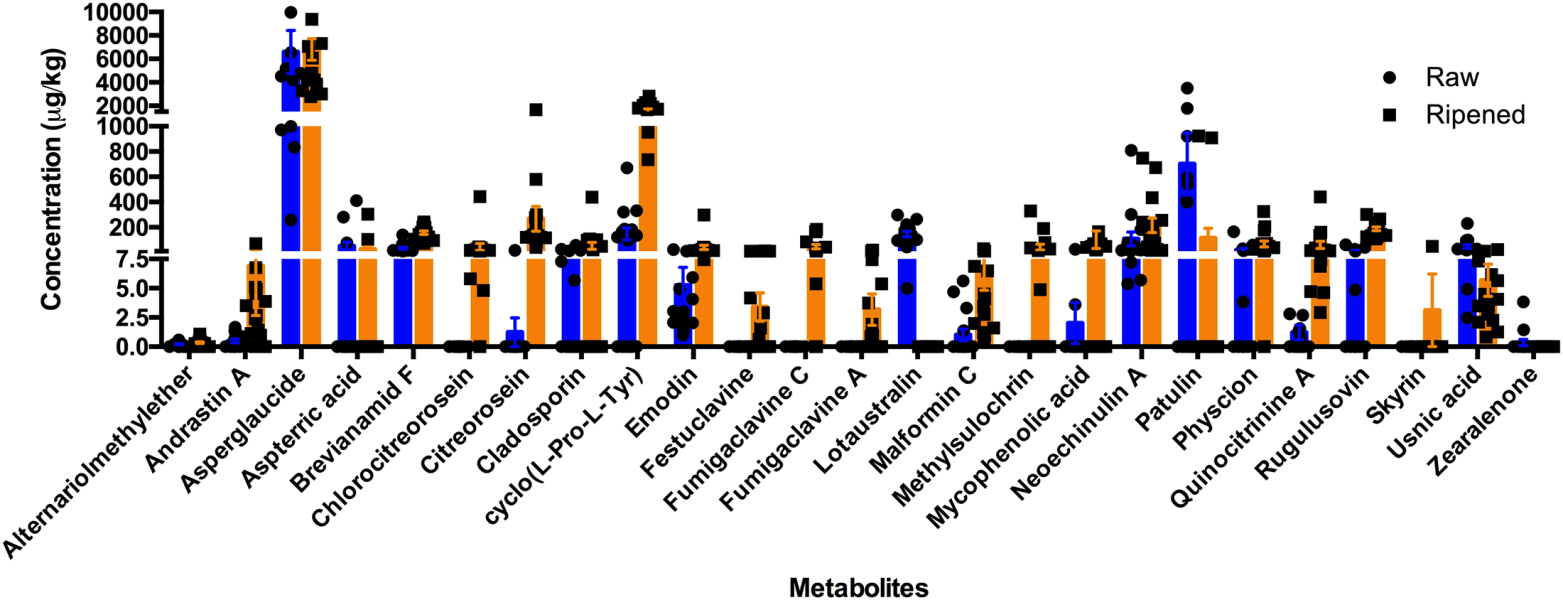
Detection of toxic metabolites in raw and ripened Pu-erh samples. Raw Pu-erh samples are indicated by circles, and ripened samples by squares. Mean concentrations and standard deviations of each metabolite in raw (in blue) and ripened (in orange) Pu-erh samples are marked.

Mycotoxin detection by LC-MS/MS was applied to all tea samples. All together 25 compounds were detected, most of them at low concentrations: alternariolmethylether, andrastin A, asperglaucide, aspterric acid, brevianamid F, chlorocitreorosein, citreorosein, cladosporin, cyclo(L-Pro-L-Tyr), emodin, festuclavine, fumigaclavine A, fumigaclavine C, lotaustralin, malformin C, methylsulochrin, mycophenolic acid, neoechinulin A, patulin, physcion, quinocitrinin, rugulusovin, skyrin, usnic acid, and zearalenone (Fig 4). Five compounds were detected in all tea samples: asperglaucide (aurantiamideacetate), brevinamide F, emodine, neoechinulin A, andusnic acid; asperglaucide was present at the highest concentration of any mycotoxin in both raw (mean concentration 6596 μg/kg) and ripened (6799 μg/kg) Pu-erh. Festuclavine, fumigaclavine A, methylsulochrin, chlorocitreorosein, and skyrin were detected in ripened samples only, while lotaustralin was found only in raw samples. Patulin was found in 9 (mean concentration 1169 μg/kg) of 15 raw samples and in just 2 (915 μg/kg) of 16 ripened samples. Cyclo(L-Pro-L-Tyr) was found at high concentrations in all ripened samples (735–2825 μg/kg), but in low concentrations only in some raw samples (76–533 μg/kg).

## Discussion

Using next generation sequencing of DNA isolated from Pu-erh, we found many more species-level OTUs, 390 fungal and 600 bacterial, than had been identified in previous studies. For example, Tian et al. used both culture-dependent and denaturing gradient gel electrophoresis methods to find *ca*. 20 fungal and 30 bacterial OTUs from 19 Pu-erh samples [22]. Zhao et al. used the dilution plating method to find 41 fungal species from 60 Pu-erh samples [21]. Several fungi have been identified in Pu-erh tea by cultivation. *Aspergillus niger*, an important industrial fungus, has been recognized as the main fermenting mold in Pu-erh production [14, 15]. We found this fungus in all raw and ripened Pu-erh samples, and its relative abundance was greater in ripened Pu-erh (second most abundant OTU, 16.1% of sequences) than in raw (15^th^ most abundant OTU, 1.4% of sequences) (Table 3). In raw Pu-erh, the most dominant fungal taxon was an undetermined *Aspergillus* sp. (24.6%). In ripened Pu-erh, the most dominant fungal species was *Blastobotrys adeninivorans* (46.5%), which, in raw Pu-erh, was the third most abundant fungus (Table 3). *B*. *adenivivorans*, another fungus of biotechnological interest [63], has been frequently isolated from Pu-erh by other researchers [15, 21]. Some fungal species frequently reported previously, such as *Penicillium chrysogenum* [21, 64] and *Yarrowia* spp.[19], were not found in our study. We cannot tell if the differences between our next generation approach and these previous studies are due to differences in fungal viability, growth rate, DNA availability, or amplification efficiency, but all four aspects are likely involved. The incomplete reference database used for taxonomy assignment is another important factor because 38% (148/390) of fungal OTUs could not be assigned to species.

One of our major findings is that fungal α-diversity is higher in fresh tea leaves than in Pu-erh and that bacterial α-diversity shows the opposite trend, that is, lower in fresh leaves than in Pu-erh (Fig 3; Figure E a-d in S1 File). With bacteria, the difference in α-diversity between leaves and tea was seen with ripened, but not raw tea (Figure E e-h in S1 File), indicating that the solid substrate fermentation was responsible for the increase.

A second major finding is that the composition of both the fungal and bacterial communities changes significantly due to Pu-erh tea fermentation, as shown by significant β-diversity differences for both fungal and bacterial communities in pairwise comparisons among fresh leaves, raw and ripened Pu-erh (Table 1). The reasons for these differences must be due to microbes present in the leaves compared to those that are acquired during processing and fermentation. Fresh leaves contain sequences representing about 54% of fungal OTUs and 22% of bacterial OTUs found in Pu-erh (Fig 1). These OTUs generally existed at a low abundance in fresh leaf and were enriched in Pu-erh. The remaining fungal and bacterial OTUs found in Pu-erh must have their origin in the manufacturing processes that allow the introduction of environmental microorganisms (e.g, pile coverings, fermentation room, and worker’s hands).

An interesting finding is that the most sought-after tea, aged raw Pu-erh, has a fungal community more like ripened than young raw Pu-erh, and a similar trend was seen for the bacterial community (Fig 3). This result indicates that the accelerated microbial fermentation of ripened Pu-erh, encouraged by the addition of water and the warmth generated by microbial fermentation, results in a microbial community composition similar to that found in much older, raw Pu-erh. It also provides an ecological explanation for the rapid acceptance and widespread use of the ripened Pu-erh process.

We sought correlations between four variables in tea production and microbial community composition, age of the tea, producer of the tea, whether the tea was pure or contaminated with other plants, and whether the tea was left loose or pressed into cakes. Age of tea showed significant correlation with fungal and bacterial community composition only for raw Pu-erh (Table C in S1 File). From this result, one might infer that raw Pu-erh is a robust but lengthy method of making the product, and that making ripened Pu-erh is a more demanding process, but one that does not benefit from aging. This result also seems to support the speculation that the long transport of raw Pu-erh from Yunnan to Tibet and other remote destinations in ancient times contributed to its maturation [8]. Aging does not significantly affect the communities of ripened tea, suggesting that aging ripened tea is unnecessary. The other three variables did not have a significant effect on microbial communities (Table C in S1 File).

Regarding our discovery in ripened but not raw Pu-erh of *rbcL* sequences from *Musa*, *Pinus*, and *Brassica* (Figure B in S1 File), we speculate that they resulted from plants or plant products used by producers to overlay tea during pile fermentation to retard water loss and retain heat [15]. Of course, contamination would also be possible from fermentation room floors, tools, or packaging materials, as well as contamination at harvest. We could not determine the amount of these contaminating plants in the ripened Pu-erh samples, but the contamination was not enough to cause a significant difference in microbial community composition (S3 Table), or the taste of brewed tea.

Although Pu-erh tea has been considered to be a safe beverage to drink for hundreds of years, with no reports of intoxication, the quality and safety of any microbially fermented product are topics of continued interest and concern. Among the compounds detected from Pu-erh in this study, the most commonly encountered was asperglaucide (Fig 4), which was detected in all samples and in high amounts in raw (6596 μg/kg) and in ripened (6799 μg/kg) tea. This metabolite is reported to be produced by *Aspergillus* spp, including *A*. *penicillioides* [65], which was detected in all samples (Table 3). It is also reported from some plants, e.g, *Walsura yunnanensis* [66], but in none of the plants detected in this study. Asperglaucide is reported to have anti-inflammatory effect and the ability to inhibit cysteine peptidases [48], which may be beneficial in protection against cartilage degeneration. Also detected in all samples was neoechinulin A, which has anti-inflammatory effects and can be produced by some *Eurotium* spp [67]. Fumigaclavine A, an antibacterial alkaloid produced by *Aspergillus* spp. [68] was detected in ripened tea only, while lotaustralin, a precursor to hydrogen cyanide [69], was detected in all samples of raw tea, but not in ripened tea samples. The fungicide cyclo(L-Pro-L-Tyr), produced by *Lysobacter capsici* [70] and *Alternaria alternata* [71], was detected in 60% and 100% of samples of raw and ripened tea, respectively, but in substantially higher amounts in ripened tea. This distribution was also the case for rugulusovin, which is produced by *Penicillium* spp. and has been shown to have cytotoxic effect against human and murine tumor cells [72–75]. It was detected in half of the raw and all of the ripened samples, a distribution that may be explained not only by differences in the microbiome, but also by differences in growth conditions between the two tea categories.

Patulin was detected in 60% of the raw samples with a mean concentration of 1169 μg/kg, and in only 12.5% of the ripened samples at a mean concentration of 915 μg/kg. Patulin is of concern because it is produced by a large number of fungi and is suspected of being clastogenic, mutagenic, teratogenic, genotoxic, and cytotoxic [76]. The US FDA has set an upper limit of 50 μg/kg for patulin in apple juice and apple juice concentrates. Although the concentration of patulin would be expected to be lower in a cup of properly prepared tea than the roughly 1000 μg/kg found by us in dry tea leaves, the patulin concentrations in prepared tea would be expected to surpass the limit set by the FDA. The discrepancy between our finding of high patulin concentration and the healthy reputation enjoyed by Pu-erh may be explained by the modulation of patulin toxicity through the action of green tea polyphenols [77].

Although we detected patulin in Pu-erh, we do not know its source. Known producers of patulin are *Penicillium* spp. (*P*. *expansum*, *P*. *griseofulvum*, *P*. *carneum*, *P*. *glandicola*, *P*. *coprobium*, *P*. *vulpinum*, *P*. *clavigenum*, and *P*. *concentricum*), *Aspergillus* spp. (*A*. *clavatus*, *A*. *giganteus*, and *A*. *terreus*), *Paecilomyces variotii*, and *Byssochlamys nivea* [78]. However, none of these species was detected in our tea samples. It is therefore likely that there are species present in the tea that have not yet been reported to produce patulin. Conversely, we found fungal species reported to produce ochratoxin A (*Aspergillus niger* in all samples and *A*. *ochraceus* in six of the ripened samples), but no ochratoxin A was detected. This result is in accordance with Mogensen et al. [24], who found no content of ochratoxin A in five Pu-erh teas investigated but different from Haas et al. [26], who detected ochratoxin A in four out of 36 Pu-erh samples. In the present study, a small amount of zearalenone was detected in one sample only, while aflatoxin, fumonisins or trichothecenes were not detected. Haas et al. did not find aflatoxins or fumonisins in the 36 Pu-erh samples tested [26]. Wu et al. investigated 70 Pu-erh samples and found that all tea samples were safe regarding fumonisin B1 and T-2 toxin, however, 8 samples displayed higher concentrations of aflatoxin B1 than the safety limit, and 63 samples exceeded the safety limit for deoxynivalenol [28]. An explanation for finding fungi capable of producing mycotoxins, but not detecting the toxins themselves, may be found in a recent report that tea extracts inhibited aflatoxin production by *Aspergillus flavus* whereas they did not inhibit mycelial growth of the fungus [79]. Inhibition of mycotoxin production without inhibiting fungal growth was also reported for plants other than tea [80]. This situation might be the case for ochratoxin, as well. As noted above, to drink Pu-erh safely, most producers or distributers of Pu-erh tea recommend discarding the first brew, a practice that may be advisable to remove water-soluble or suspended contaminants.

## Conclusions

Next generation sequencing revealed high fungal and bacterial diversity in Pu-erh tea. Fungal diversity drops and bacterial diversity rises as a result of raw or ripened fermentation. The composition of microbial communities changes significantly among fresh leaves, raw and ripened Pu-erh with the aged raw tea having similar community to ripened tea. Age of tea is identified as a significant variable affecting microbial community of raw tea, but not of ripened tea. Multiple mycotoxins were detected from either or both categories of Pu-erh, but all but patulin and asperglaucide were under the safety limit. For safe drinking, we recommend discarding the first brew.

## Supporting Information

S1 File Figure A in S1 File. Raw and ripened Pu-erh display differences on both tea appearance (a, c) and the color of infusion (b). Figure B in S1 File. Illustration of *rbcL* sequencing results from samples used in this study. Figure C in S1 File. Proportion of chloroplast sequences within each sample in the bacterial SSU dataset. Figure D in S1 File. Relative proportions of OTUs/sequences assigned to each fungal/bacterial phylum. Figure E in S1 File. α-diversity comparisons among fresh leaves, raw and ripened Pu-erh samples on different α-diversity indices. Figure F in S1 File. Ordination (nonmetric multidimensional scaling; NMDS) of microbial community structure (Bray-Curtis dissimilarity) on fresh tea leaf (in black), raw Pu-erh (in blue), and ripened Pu-erh (in red). Table A in S1 File. Metadata used in this study. Table B in S1 File. Fungal and bacterial indicator taxa detected for fresh tea leaf, raw Pu-erh, ripened Pu-erh, and raw+ripened Pu-erh. Table C in S1 File. ANOSIM and ADONIS test of four variables on fungal/bacterial community in raw/ripened Pu-erh. Table D in S1 File. Mantel test between the fungal and bacterial communities based on either Binary-Jaccard or Bray-Curtis distance matrices. Table E in S1 File. The first 15 most abundant bacterial OTUs in fresh leaf, raw and ripened Pu-erh samples.
(PDF)
