# Supplementary material for: The Microbiome and Metabolites in Fermented Pu-erh Tea as Revealed by High-Throughput Sequencing and Quantitative Multiplex Metabolite Analysis
Source: PLoS One. 2016 Jun 23;11(6):e0157847. doi: 10.1371/journal.pone.0157847 (PMC4918958; doi:10.1371/journal.pone.0157847)
Supplement: S1 File — Figure A in S1 File. Raw and ripened Pu-erh display differences on both tea appearance (a, c) and the color of infusion (b). Figure B in S1 File. Illustration of rbcL sequencing results from samples used in this study. Figure C in S1 File. Proportion of chloroplast sequences within each sample in the bacterial SSU dataset. Figure D in S1 File. Relative proportions of OTUs/sequences assigned to each fungal/bacterial phylum. Figure E in S1 File. α-diversity comparisons among fresh leaves, raw and ripened Pu-erh samples on different α-diversity indices. Figure F in S1 File. Ordination (nonmetric multidimensional scaling; NMDS) of microbial community structure (Bray-Curtis dissimilarity) on fresh tea leaf (in black), raw Pu-erh (in blue), and ripened Pu-erh (in red). Table A in S1 File. Metadata used in this study. Table B in S1 File. Fungal and bacterial indicator taxa detected for fresh tea leaf, raw Pu-erh, ripened Pu-erh, and raw+ripened Pu-erh. Table C in S1 File. ANOSIM and ADONIS test of four variables on fungal/bacterial community in raw/ripened Pu-erh. Table D in S1 File. Mantel test between the fungal and bacterial communities based on either Binary-Jaccard or Bray-Curtis distance matrices. Table E in S1 File. The first 15 most abundant bacterial OTUs in fresh leaf, raw and ripened Pu-erh samples. (PDF) [file pone.0157847.s001.pdf]

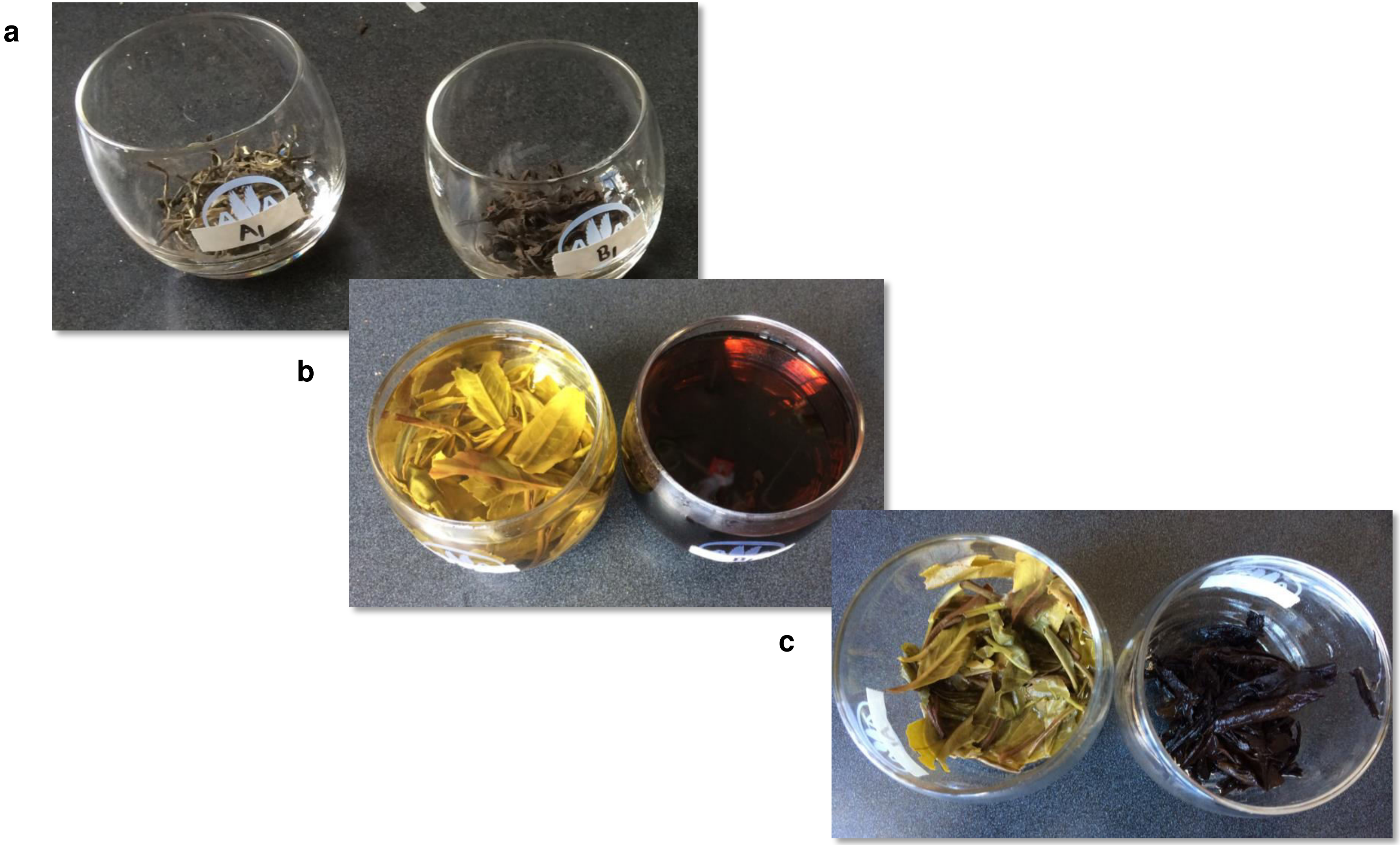

Figure A. Raw and ripened Pu-erh display differences on both tea appearance (a, c) and the color of infusion (b). Ripened Pu-erh is generally darker than raw Pu-erh due to the “pile fermentation” involved during its manufacturing process. a, before adding water; b, with water added; c, after discarding the brew. In each subpanel, the left cup represents raw Pu-erh and the right represents ripened Pu-erh. For each type, young Pu-erh tea with the same age was illustrated.

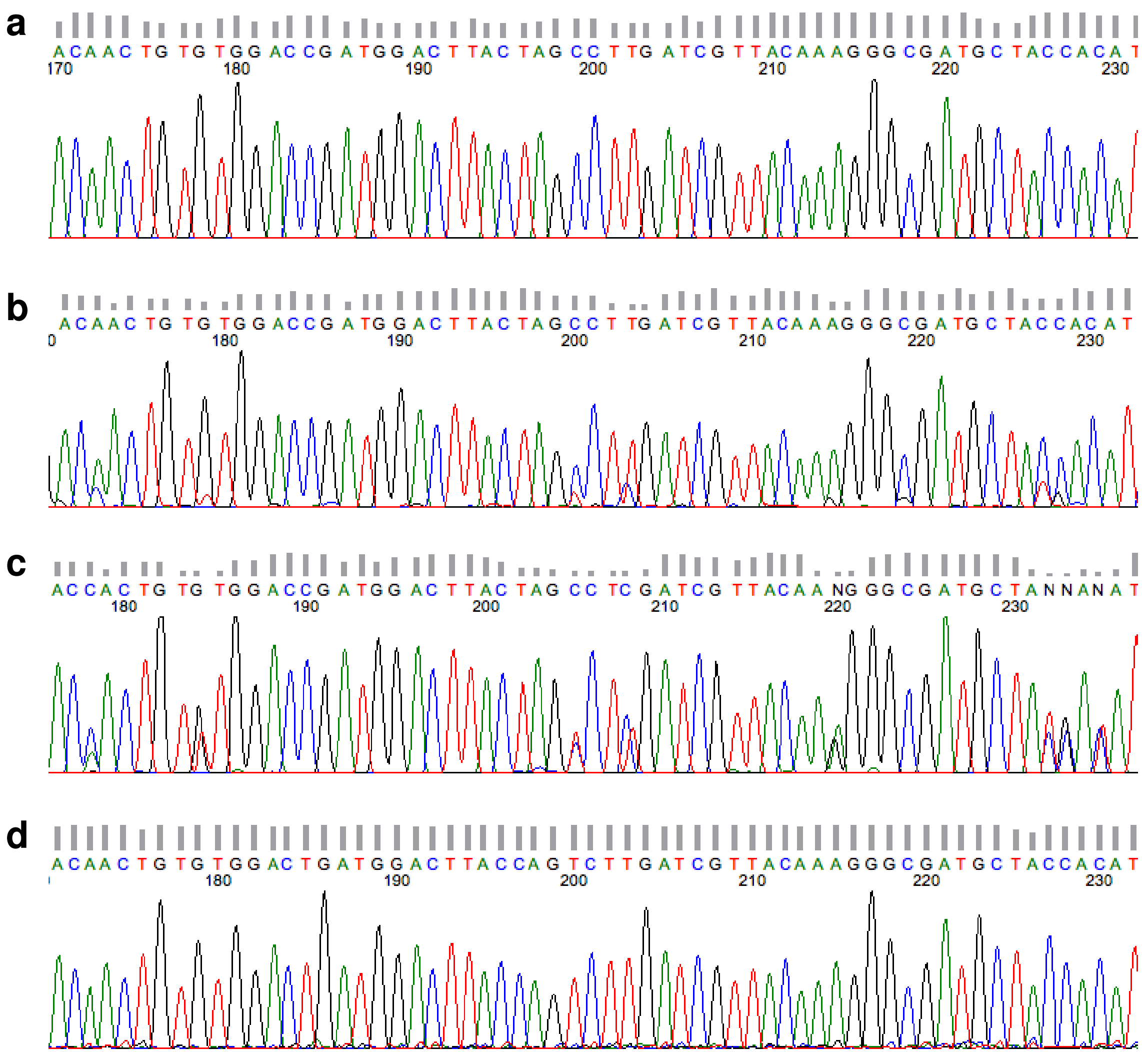

Figure B. Illustration of *rbcL* sequencing results from samples used in this study. All fresh leaf and raw Pu-erh samples and most ripened Pu-erh samples showed pure sequences of the tea plant *Camellia sinensis* (a). Several ripened Pu-erh samples, however, showed heterozygous peaks at certain nucleotide sites, but nucleotides characteristic of *C. sinensis* could always be found (b and c). Three ripened Pu-erh samples had homozygous chromatogram during our initial trial, but they actually represented *non-C. sinensis* plants (d); true *C. sinensis rbcL* sequence were later detected from these samples using newly extracted DNA solutions.

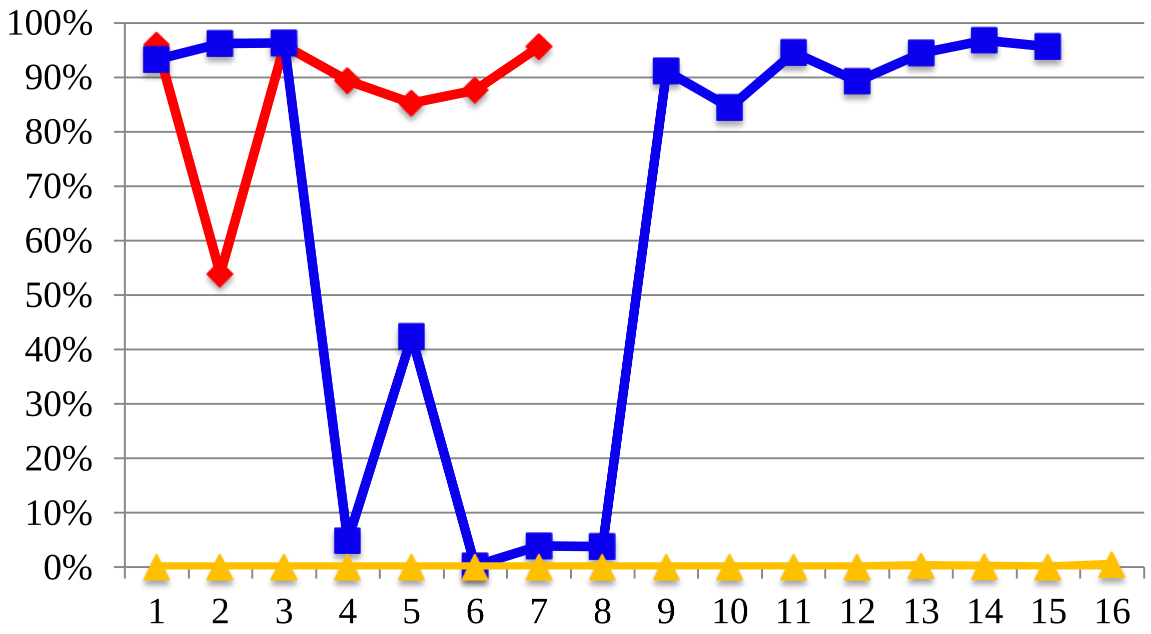

Figure C. Proportion of chloroplast sequences within each sample in the bacterial 16S dataset. The x-axis represents different samples of each sample type (i.e., fresh tea leaf, raw Pu-erh and ripened Pu-erh) in the same order as Table S1. Ripened Pu-erh samples (in orange) had only few chloroplast sequences, but fresh tea leaf samples (in red) and raw Pu-erh samples (in blue) contained a great amount of chloroplast sequences. A low proportion of chloroplast sequences were also detected in some aged raw Pu-erh samples (e.g., A6, A7, A8).

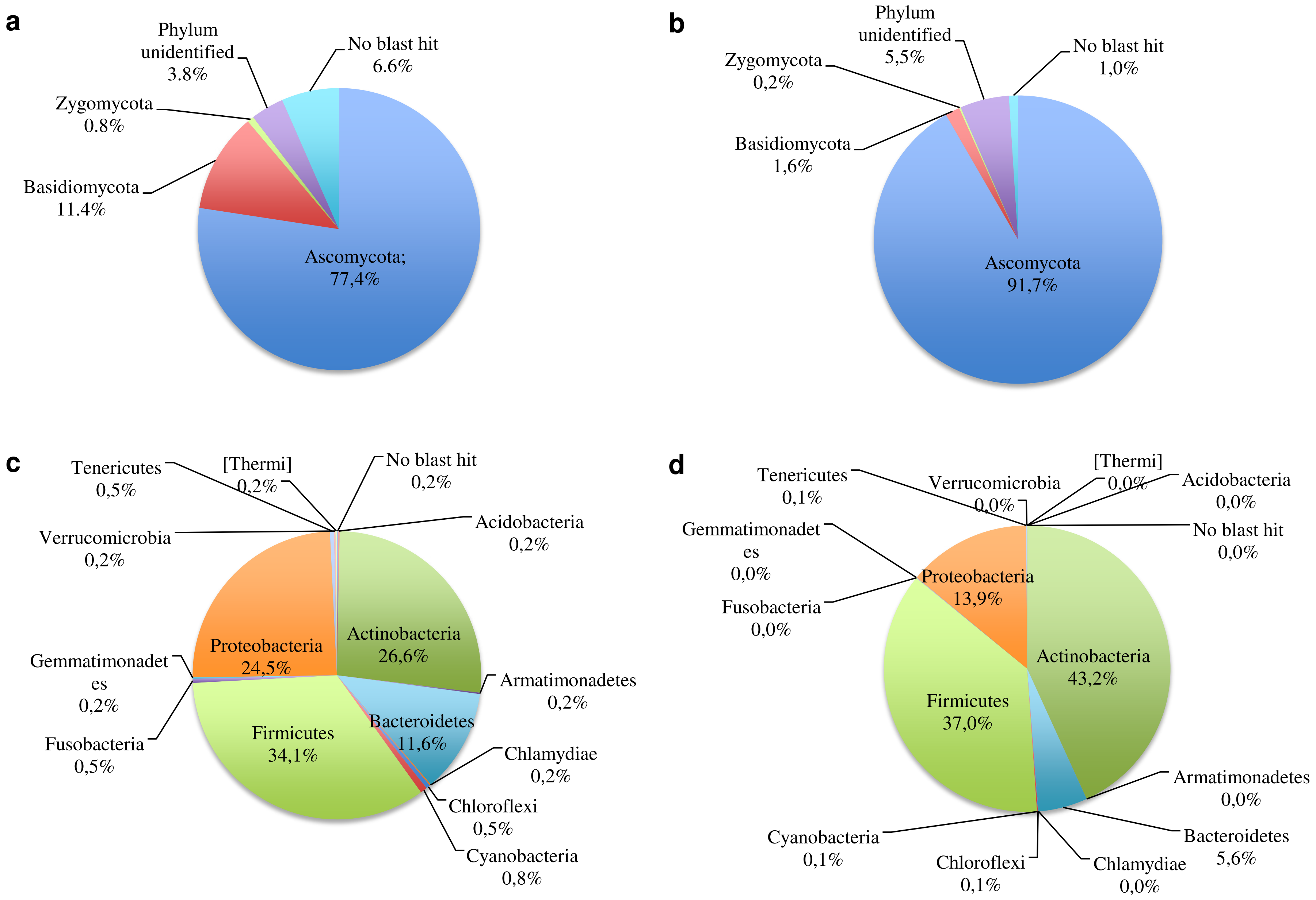

Figure D. Relative proportions of OTUs/sequences assigned to each fungal/bacterial phylum. For fungi, Ascomycota is most abundant according to either OTUs (a) or sequences (b). For bacteria, Actinobacteria, Firmicutes, and Proteobacteria are more dominant than other orders according to either OTUs (c) or sequences (d).

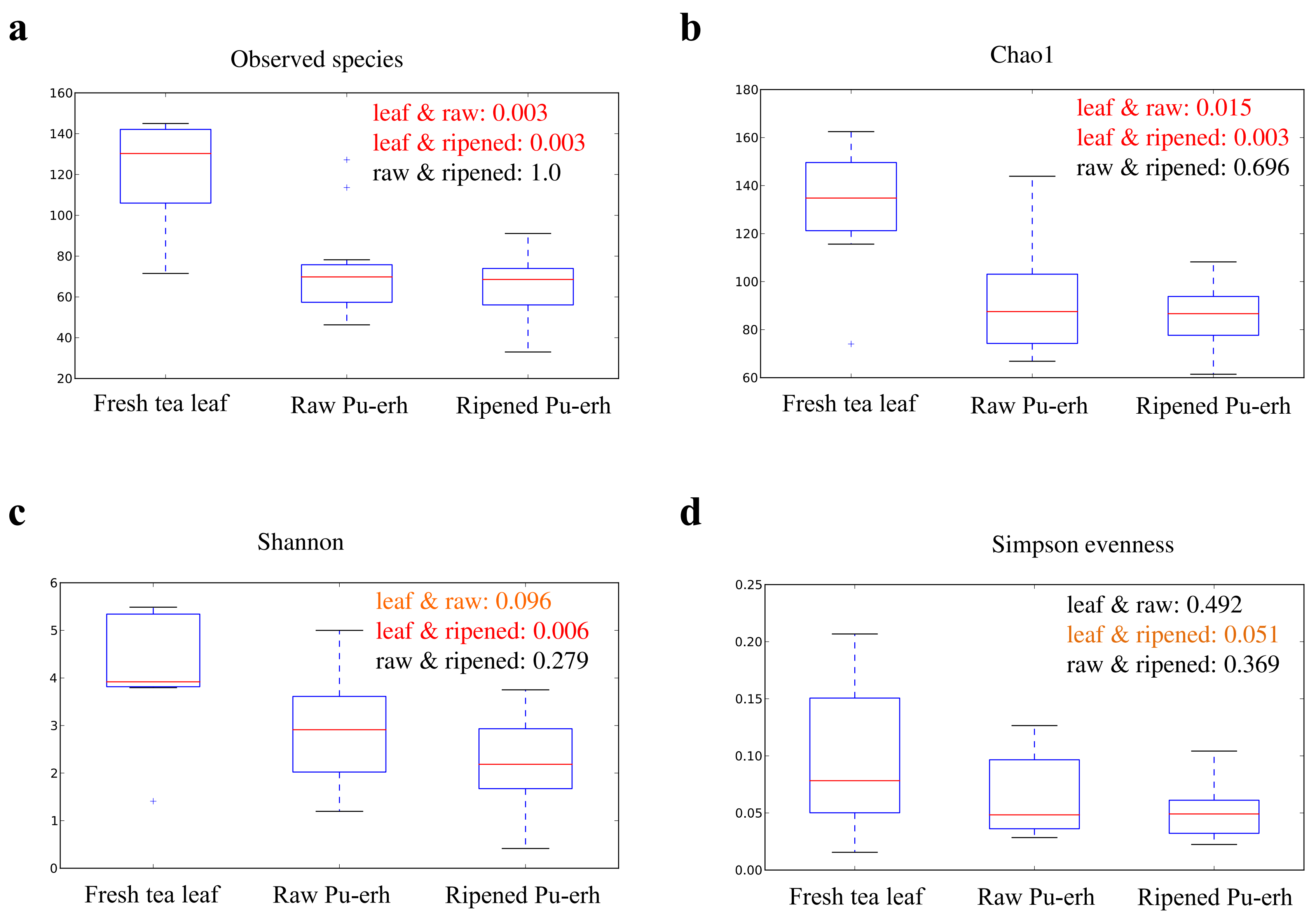
a
Observed species
leaf & raw: 0.003
leaf & ripened: 0.003
raw & ripened: 1.0
160
140
120
100
80
60
40
20
Fresh tea leaf
Raw Pu-erh
Ripened Pu-erh
b
Chao1
leaf & raw: 0.015
leaf & ripened: 0.003
raw & ripened: 0.696
180
160
140
120
100
80
60
Fresh tea leaf
Raw Pu-erh
Ripened Pu-erh
c
Shannon
leaf & raw: 0.096
leaf & ripened: 0.006
raw & ripened: 0.279
6
5
4
3
2
1
0
Fresh tea leaf
Raw Pu-erh
Ripened Pu-erh
d
Simpson evenness
leaf & raw: 0.492
leaf & ripened: 0.051
raw & ripened: 0.369
0.25
0.20
0.15
0.10
0.05
0.00
Fresh tea leaf
Raw Pu-erh
Ripened Pu-erh

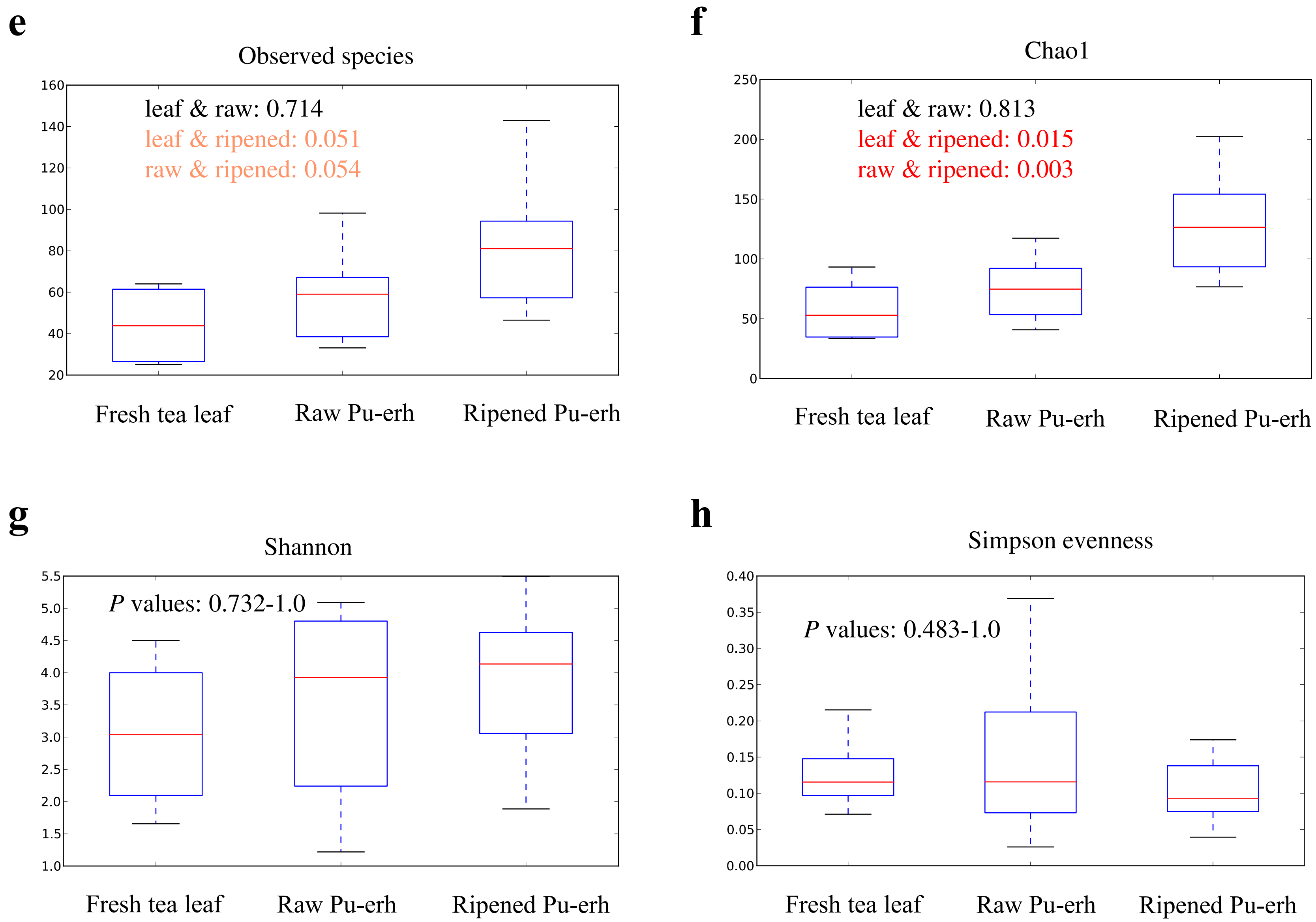

Figure E. Alpha-diversity comparisons among fresh leaves, raw and ripened Pu-erh samples on different alpha-diversity indices. a-d, fungi; e-h, bacteria. *P*-values were given for pairwise comparisons.

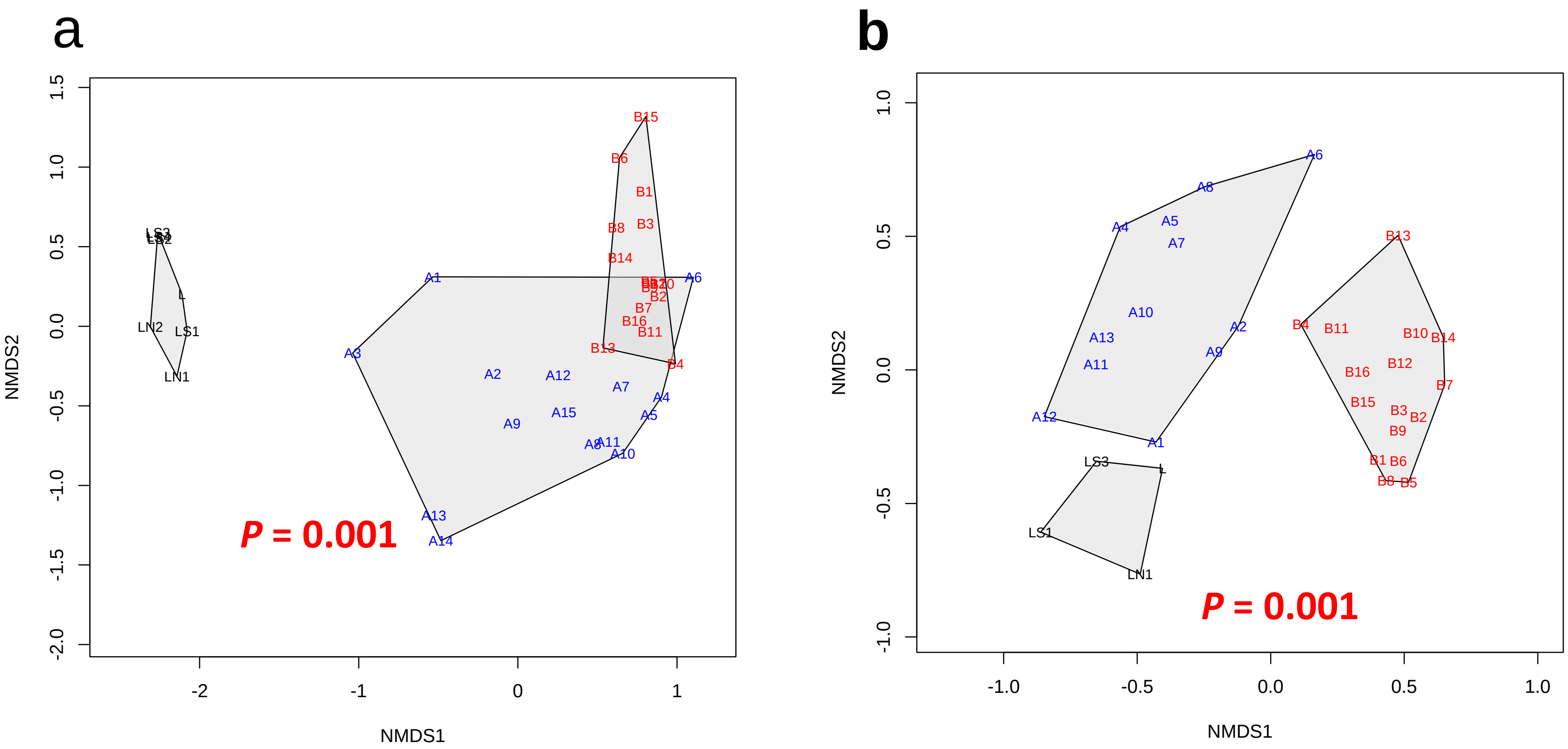

Figure F. Ordination (nonmetric multidimensional scaling; NMDS) of microbial community structure (Bray–Curtis dissimilarity) on fresh tea leaf (in black), raw raw Pu-erh (in blue), and ripened Pu-erh (in red). They were significantly different (*P* = 0.001) at both fungal (a) and bacterial (b) community structures.

Table A. Metadata used in this study

Sample ID	Sample type	Age (year)	Age stage [a]	Producer [b]	Plant status [c]	Tea form [d]
L	fresh tea leaf	NA	NA	NA	pure	NA
LN1	fresh tea leaf	NA	NA	NA	pure	NA
LN2	fresh tea leaf	NA	NA	NA	pure	NA
LS1	fresh tea leaf	NA	NA	NA	pure	NA
LS2	fresh tea leaf	NA	NA	NA	pure	NA
LS3	fresh tea leaf	NA	NA	NA	pure	NA
LS4	fresh tea leaf	NA	NA	NA	pure	NA
A1	raw Pu-erh	0	Young	Com1	pure	Loose
A2	raw Pu-erh	1	Young	Com1	pure	Loose
A3	raw Pu-erh	2	Young	Com1	pure	Loose
A4	raw Pu-erh	5	Middle	Com1	pure	Loose
A5	raw Pu-erh	10	Old	Com1	pure	Loose
A6	raw Pu-erh	28	Old	Com2	pure	Compressd
A7	raw Pu-erh	6	Middle	Com2	pure	Compressd
A8	raw Pu-erh	11	Old	Com2	pure	Compressd
A9	raw Pu-erh	1	Young	Com2	pure	Compressd
A10	raw Pu-erh	7	Middle	Com3	pure	Compressd
A11	raw Pu-erh	5	Middle	Com3	pure	Compressd
A12	raw Pu-erh	6	Middle	Com4	pure	Compressd
A13	raw Pu-erh	2	Young	Com4	pure	Compressd
A14	raw Pu-erh	2	Young	Com4	pure	Loose
A15	raw Pu-erh	5	Middle	Com5	pure	Compressd
B1	ripened Pu-erh	0	Young	Com1	pure	Loose
B2	ripened Pu-erh	1	Young	Com1	mix	Loose
B3	ripened Pu-erh	2	Young	Com1	pure	Loose
B4	ripened Pu-erh	5	Young	Com1	mix	Loose
B5	ripened Pu-erh	7	Old	Com2	pure	Loose
B6	ripened Pu-erh	5	Young	Com2	pure	Loose
B7	ripened Pu-erh	4	Young	Com2	mix	Loose
B8	ripened Pu-erh	3	Young	Com2	mix	Loose
B9	ripened Pu-erh	8	Old	Com3	mix	Loose
B10	ripened Pu-erh	7	Old	Com3	pure	Compressd
B11	ripened Pu-erh	6	Old	Com3	mix	Loose
B12	ripened Pu-erh	6	Old	Com3	pure	Loose
B13	ripened Pu-erh	13	Old	Com4	mix	Compressd
B14	ripened Pu-erh	5	Young	Com4	pure	Compressd
B15	ripened Pu-erh	4	Young	Com4	pure	Compressd
B16	ripened Pu-erh	3	Young	Com4	pure	Compressd

[a] According to age distribution of samples, raw Pu-erh samples were binned into three age stages (young, middle aged, and old) and ripened Pu-erh samples into two (young and old).

[b] From a producer, both raw and ripened Pu-erh samples were generally obtained.

[c] "Plant status" is determinbed by whether non-*Camellia sinensis rbcL* sequences were detected from Sanger sequencing. Pure, only sequence of *C. sinensis* was detected; mix, sequences of both *C. sinensis* and other plants were obtained.

[d] "Tea form" means loose tea or compressed tea which is generally in the cake or brick form.

Table B. Fungal and bacterial indicator taxa detected for fresh tea leaf, raw Pu-erh, ripened Pu-erh, and raw+ripened Pu-erh

Fungi	Fresh tea leaf	
	OTU_327	k__Fungi;p__Ascomycota;c__Dothideomycetes;o__Pleosporales;f__Cucurbitariaceae;g__Pyrenochaetopsis;s__Pyrenochaetopsis sp
	OTU_321	No blast hit
	OTU_320	k__Fungi;p__Ascomycota;c__Dothideomycetes;o__Pleosporales;f__Massarinaceae;g__unidentified;s__Massarinaceae sp
	OTU_323	k__Fungi;p__Basidiomycota;c__Microbotryomycetes;o__Sporidiobolales;f__Incertae sedis;g__Rhodotorula;s__Rhodotorula lamellibrachiae
	OTU_486	k__Fungi;p__unidentified;c__unidentified;o__unidentified;f__unidentified;g__unidentified;s__Fungi sp
	OTU_328	k__Fungi;p__Ascomycota;c__Dothideomycetes;o__Dothideales;f__Dothioraceae;g__Aureobasidium;s__Aureobasidium sp SWP_2012
	OTU_247	k__Fungi;p__Ascomycota;c__Sordariomycetes;o__unidentified;f__unidentified;g__unidentified;s__Sordariomycetes sp
	OTU_244	k__Fungi;p__Basidiomycota;c__Tremellomycetes;o__Tremellales;f__Incertae sedis;g__Cryptococcus;s__Cryptococcus sp QMW_2009a
	OTU_245	k__Fungi;p__Ascomycota;c__Dothideomycetes;o__Pleosporales;f__Leptosphaeriaceae;g__Coniothyrium;s__Coniothyrium sp JK27
	OTU_1789	k__Fungi;p__Ascomycota;c__Dothideomycetes;o__Capnodiales;f__Mycosphaerellaceae;g__Passalora;s__Passalora sp CBS 113378
	OTU_392	k__Fungi;p__Ascomycota;c__unidentified;o__unidentified;f__unidentified;g__unidentified;s__Ascomycota sp
	OTU_1459	k__Fungi;p__Ascomycota;c__Eurotiomycetes;o__Chaetothyriales;f__Chaetothyriaceae;g__unidentified;s__Chaetothyriaceae sp
	OTU_556	No blast hit
	OTU_129	k__Fungi;p__Basidiomycota;c__Microbotryomycetes;o__Sporidiobolales;f__Incertae sedis;g__Rhodotorula;s__Rhodotorula aurantiaca
	OTU_127	k__Fungi;p__Ascomycota;c__Dothideomycetes;o__Pleosporales;f__unidentified;g__unidentified;s__Pleosporales sp
	OTU_126	k__Fungi;p__Ascomycota;c__Sordariomycetes;o__Hypocreales;f__Nectriaceae;g__Gibberella;s__Gibberella zeae
	OTU_159	k__Fungi;p__Ascomycota;c__unidentified;o__unidentified;f__unidentified;g__unidentified;s__Ascomycota sp
	OTU_69	k__Fungi;p__Ascomycota;c__Sordariomycetes;o__Incertae sedis;f__Glomerellaceae;g__Colletotrichum;s__Colletotrichum nymphaeae
	OTU_68	k__Fungi;p__Ascomycota;c__Sordariomycetes;o__Incertae sedis;f__Glomerellaceae;g__Colletotrichum;s__Colletotrichum xanthorrhoeae
	OTU_64	k__Fungi;p__Ascomycota;c__Dothideomycetes;o__Pleosporales;f__Incertae sedis;g__unidentified;s__Pleosporales sp
	OTU_67	k__Fungi;p__unidentified;c__unidentified;o__unidentified;f__unidentified;g__unidentified;s__Fungi sp
	OTU_338	No blast hit
	OTU_238	k__Fungi;p__Ascomycota;c__Dothideomycetes;o__Pleosporales;f__Incertae sedis;g__Peyronellaea;s__Peyronellaea sancta
	OTU_233	k__Fungi;p__Ascomycota;c__Dothideomycetes;o__Capnodiales;f__Teratosphaeriaceae;g__Teratosphaeria;s__Teratosphaeria knoxdavesii
	OTU_231	k__Fungi;p__Ascomycota;c__Dothideomycetes;o__Pleosporales;f__Incertae sedis;g__Periconia;s__Periconia sp
	OTU_1484	k__Fungi;p__Ascomycota;c__unidentified;o__unidentified;f__unidentified;g__unidentified;s__Ascomycota sp
	OTU_340	k__Fungi;p__Ascomycota;c__Dothideomycetes;o__Capnodiales;f__Mycosphaerellaceae;g__Pseudocercospora;s__Pseudocercospora pyracanthigena
	OTU_427	k__Fungi;p__Ascomycota;c__Incertae sedis;o__Incertae sedis;f__Incertae sedis;g__Knufia;s__Knufia sp
	OTU_61	k__Fungi;p__Ascomycota;c__Dothideomycetes;o__Pleosporales;f__unidentified;g__unidentified;s__Pleosporales sp
	OTU_1891	k__Fungi;p__Ascomycota;c__Eurotiomycetes;o__Chaetothyriales;f__Incertae sedis;g__Strelitziana;s__Strelitziana africana
	OTU_1387	k__Fungi;p__unidentified;c__unidentified;o__unidentified;f__unidentified;g__unidentified;s__Fungi sp
	OTU_194	k__Fungi;p__Ascomycota;c__Dothideomycetes;o__Pleosporales;f__Phaeosphaeriaceae;g__unidentified;s__Phaeosphaeriaceae sp
	OTU_251	k__Fungi;p__Ascomycota;c__Dothideomycetes;o__Myriangiales;f__unidentified;g__unidentified;s__Myriangiales sp
	OTU_191	k__Fungi;p__Ascomycota;c__Sordariomycetes;o__Incertae sedis;f__Glomerellaceae;g__unidentified;s__Glomerellaceae sp
	OTU_58	k__Fungi;p__Ascomycota;c__Sordariomycetes;o__Incertae sedis;f__Plectosphaerellaceae;g__Plectosphaerella;s__Plectosphaerella citrulli
	OTU_59	k__Fungi;p__Ascomycota;c__Dothideomycetes;o__Pleosporales;f__Phaeosphaeriaceae;g__Phaeoseptoria;s__Phaeoseptoria sp FF_2011
	OTU_50	k__Fungi;p__Ascomycota;c__Sordariomycetes;o__Xylariales;f__Amphisphaeriaceae;g__Pestalotiopsis;s__Pestalotiopsis foedans
	OTU_54	k__Fungi;p__Basidiomycota;c__Tremellomycetes;o__Tremellales;f__Incertae sedis;g__Hannaella;s__Hannaella luteola
	OTU_55	k__Fungi;p__Ascomycota;c__Eurotiomycetes;o__Chaetothyriales;f__Incertae sedis;g__Strelitziana;s__Strelitziana mali
	OTU_228	No blast hit
	OTU_185	k__Fungi;p__Basidiomycota;c__Microbotryomycetes;o__Sporidiobolales;f__Incertae sedis;g__Rhodotorula;s__Rhodotorula graminis
	OTU_90	k__Fungi;p__Ascomycota;c__Dothideomycetes;o__Capnodiales;f__Mycosphaerellaceae;g__Passalora;s__Passalora loranthi
	OTU_99	k__Fungi;p__Basidiomycota;c__Tremellomycetes;o__Tremellales;f__Incertae sedis;g__Cryptococcus;s__Cryptococcus sp CBS 8358
	OTU_1416	k__Fungi;p__Ascomycota;c__Dothideomycetes;o__Pleosporales;f__Phaeosphaeriaceae;g__Stagonospora;s__Stagonospora sp AX113
	OTU_267	k__Fungi;p__Ascomycota;c__Eurotiomycetes;o__Chaetothyriales;f__unidentified;g__unidentified;s__Chaetothyriales sp
	OTU_268	k__Fungi;p__Basidiomycota;c__Microbotryomycetes;o__Sporidiobolales;f__Incertae sedis;g__Rhodotorula;s__Rhodotorula sp 3_23T
	OTU_269	k__Fungi;p__Ascomycota;c__Dothideomycetes;o__Capnodiales;f__Mycosphaerellaceae;g__unidentified;s__Mycosphaerellaceae sp
	OTU_174	k__Fungi;p__Ascomycota;c__Taphrinomycetes;o__Taphrinales;f__Taphrinaceae;g__Lalaria;s__Lalaria inositophila
	OTU_188	k__Fungi;p__Ascomycota;c__Dothideomycetes;o__Capnodiales;f__Mycosphaerellaceae;g__Septoria;s__Septoria glycinicola
	OTU_176	k__Fungi;p__Ascomycota;c__Dothideomycetes;o__Pleosporales;f__Phaeosphaeriaceae;g__Stagonospora;s__Stagonospora sp AX113
	OTU_101	k__Fungi;p__Ascomycota;c__Dothideomycetes;o__Capnodiales;f__Mycosphaerellaceae;g__unidentified;s__Mycosphaerellaceae sp
	OTU_103	k__Fungi;p__Ascomycota;c__Sordariomycetes;o__Hypocreales;f__Nectriaceae;g__unidentified;s__Nectriaceae sp
	OTU_106	k__Fungi;p__Basidiomycota;c__Tremellomycetes;o__Tremellales;f__Incertae sedis;g__unidentified;s__Tremellales sp
	OTU_46	k__Fungi;p__Ascomycota;c__Dothideomycetes;o__Pleosporales;f__Incertae sedis;g__Phoma;s__Phoma sp P32E1
	OTU_40	k__Fungi;p__unidentified;c__unidentified;o__unidentified;f__unidentified;g__unidentified;s__Fungi sp
	OTU_49	k__Fungi;p__Ascomycota;c__Dothideomycetes;o__Pleosporales;f__unidentified;g__unidentified;s__Pleosporales sp
	OTU_48	k__Fungi;p__Basidiomycota;c__Microbotryomycetes;o__Sporidiobolales;f__Incertae sedis;g__Sporobolomyces;s__Sporobolomyces oryzicola
	OTU_196	k__Fungi;p__Ascomycota;c__Sordariomycetes;o__Hypocreales;f__Incertae sedis;g__Sarocladium;s__Sarocladium strictum
	OTU_410	k__Fungi;p__Basidiomycota;c__unidentified;o__unidentified;f__unidentified;g__unidentified;s__Basidiomycota sp
	OTU_362	k__Fungi;p__Ascomycota;c__Dothideomycetes;o__Pleosporales;f__Cucurbitariaceae;g__Curreya;s__Curreya sp
	OTU_364	No blast hit
	OTU_366	k__Fungi;p__Ascomycota;c__unidentified;o__unidentified;f__unidentified;g__unidentified;s__Ascomycota sp
	OTU_113	k__Fungi;p__Basidiomycota;c__Tremellomycetes;o__unidentified;f__unidentified;g__unidentified;s__Tremellomycetes sp
	OTU_85	k__Fungi;p__Ascomycota;c__Dothideomycetes;o__Pleosporales;f__Incertae sedis;g__Phoma;s__Phoma sp FF_2011

OTU_1120	k__Fungi;p__Ascomycota;c__Sordariomycetes;o__Xylariales;f__Amphisphaeriaceae;g__Pestalotiopsis;s__Pestalotiopsis rosea
OTU_843	k__Fungi;p__Ascomycota;c__Dothideomycetes;o__Pleosporales;f__Incertae sedis;g__Phoma;s__Phoma tropica
OTU_119	k__Fungi;p__Ascomycota;c__Sordariomycetes;o__Xylariales;f__Amphisphaeriaceae;g__Pestalotiopsis;s__Pestalotiopsis trachicarpicola
OTU_270	k__Fungi;p__Ascomycota;c__unidentified;o__unidentified;f__unidentified;g__unidentified;s__Ascomycota sp
OTU_1852	k__Fungi;p__Ascomycota;c__Dothideomycetes;o__Pleosporales;f__Phaeosphaeriaceae;g__unidentified;s__Phaeosphaeriaceae sp
OTU_215	k__Fungi;p__Ascomycota;c__Dothideomycetes;o__Pleosporales;f__Leptosphaeriaceae;g__Coniothyrium;s__Coniothyrium sidae
OTU_214	k__Fungi;p__Ascomycota;c__Lecanoromycetes;o__Pertusariales;f__Ochrolechiaceae;g__Ochrolechia;s__Ochrolechia frigida
OTU_1677	k__Fungi;p__Basidiomycota;c__unidentified;o__unidentified;f__unidentified;g__unidentified;s__Basidiomycota sp
OTU_213	k__Fungi;p__Ascomycota;c__Dothideomycetes;o__Capnodiales;f__Mycosphaerellaceae;g__Pseudocercospora;s__Pseudocercospora robusta
OTU_212	k__Fungi;p__Ascomycota;c__Sordariomycetes;o__Xylariales;f__Amphisphaeriaceae;g__Pestalotiopsis;s__Pestalotiopsis diversiseta
OTU_32	k__Fungi;p__Ascomycota;c__unidentified;o__unidentified;f__unidentified;g__unidentified;s__Ascomycota sp
OTU_34	k__Fungi;p__Ascomycota;c__Dothideomycetes;o__Dothideales;f__Dothioraceae;g__Selenophoma;s__Selenophoma mahoniae
OTU_200	k__Fungi;p__Ascomycota;c__Eurotiomycetes;o__Verrucariales;f__Verrucariaceae;g__Placidiopsis;s__Placidiopsis cinerascens
OTU_207	k__Fungi;p__Basidiomycota;c__Tremellomycetes;o__Tremellales;f__Incertae sedis;g__unidentified;s__Tremellales sp
OTU_204	k__Fungi;p__Ascomycota;c__unidentified;o__unidentified;f__unidentified;g__unidentified;s__Ascomycota sp
OTU_299	k__Fungi;p__Ascomycota;c__Dothideomycetes;o__Capnodiales;f__Davidiellaceae;g__Cladosporium;s__Cladosporium langeronii
OTU_110	k__Fungi;p__Basidiomycota;c__Microbotryomycetes;o__Sporidiobolales;f__Incertae sedis;g__Rhodotorula;s__Rhodotorula lactosa
OTU_111	k__Fungi;p__Ascomycota;c__Dothideomycetes;o__Incertae sedis;f__Incertae sedis;g__Leptospora;s__Leptospora rubella
OTU_292	k__Fungi;p__Ascomycota;c__Dothideomycetes;o__Capnodiales;f__Mycosphaerellaceae;g__Sphaerulina;s__Sphaerulina tirolensis
OTU_476	k__Fungi;p__Ascomycota;c__Leotiomycetes;o__Helotiales;f__unidentified;g__unidentified;s__Helotiales sp
OTU_471	k__Fungi;p__Ascomycota;c__Eurotiomycetes;o__Chaetothyriales;f__Herpotrichiellaceae;g__Capronia;s__Capronia pilosella
OTU_277	k__Fungi;p__Ascomycota;c__Dothideomycetes;o__Incertae sedis;f__Incertae sedis;g__Leptospora;s__Leptospora rubella
OTU_276	k__Fungi;p__Ascomycota;c__Dothideomycetes;o__Capnodiales;f__Mycosphaerellaceae;g__Stenella;s__Stenella araguata
OTU_274	k__Fungi;p__Ascomycota;c__Dothideomycetes;o__Pleosporales;f__Incertae sedis;g__Ascochyta;s__Ascochyta maackiae
OTU_279	No blast hit
OTU_139	k__Fungi;p__Ascomycota;c__Dothideomycetes;o__Pleosporales;f__unidentified;g__unidentified;s__Pleosporales sp
OTU_135	k__Fungi;p__Basidiomycota;c__unidentified;o__unidentified;f__unidentified;g__unidentified;s__Basidiomycota sp
OTU_130	k__Fungi;p__Ascomycota;c__unidentified;o__unidentified;f__unidentified;g__unidentified;s__Ascomycota sp
OTU_190	k__Fungi;p__Ascomycota;c__Sordariomycetes;o__Hypocreales;f__Nectriaceae;g__Fusarium;s__Fusarium sp IBL 03157
OTU_1916	k__Fungi;p__Ascomycota;c__Dothideomycetes;o__Pleosporales;f__Phaeosphaeriaceae;g__unidentified;s__Phaeosphaeriaceae sp
OTU_20	k__Fungi;p__Ascomycota;c__Dothideomycetes;o__Pleosporales;f__Incertae sedis;g__Phoma;s__Phoma infossa
OTU_25	k__Fungi;p__Ascomycota;c__Dothideomycetes;o__Capnodiales;f__Mycosphaerellaceae;g__Septoria;s__Septoria aegopodina
OTU_24	No blast hit
OTU_29	k__Fungi;p__Basidiomycota;c__Tremellomycetes;o__unidentified;f__unidentified;g__unidentified;s__Tremellomycetes sp
OTU_95	k__Fungi;p__Ascomycota;c__unidentified;o__unidentified;f__unidentified;g__unidentified;s__Ascomycota sp
OTU_167	k__Fungi;p__Ascomycota;c__Eurotiomycetes;o__Chaetothyriales;f__Incertae sedis;g__Strelitziana;s__Strelitziana mali
OTU_166	k__Fungi;p__Ascomycota;c__Dothideomycetes;o__Capnodiales;f__unidentified;g__unidentified;s__Capnodiales sp
OTU_287	k__Fungi;p__Basidiomycota;c__Tremellomycetes;o__Tremellales;f__Incertae sedis;g__Hannaella;s__Hannaella kunmingensis
OTU_264	k__Fungi;p__unidentified;c__unidentified;o__unidentified;f__unidentified;g__unidentified;s__Fungi sp
OTU_309	k__Fungi;p__Ascomycota;c__Dothideomycetes;o__Capnodiales;f__Mycosphaerellaceae;g__Mycosphaerella;s__Mycosphaerella yunnanensis
OTU_308	k__Fungi;p__Ascomycota;c__unidentified;o__unidentified;f__unidentified;g__unidentified;s__Ascomycota sp
OTU_303	k__Fungi;p__Basidiomycota;c__Tremellomycetes;o__unidentified;f__unidentified;g__unidentified;s__Tremellomycetes sp
OTU_305	k__Fungi;p__Ascomycota;c__Dothideomycetes;o__Myriangiales;f__unidentified;g__unidentified;s__Myriangiales sp
OTU_224	No blast hit
OTU_1438	k__Fungi;p__Ascomycota;c__unidentified;o__unidentified;f__unidentified;g__unidentified;s__Ascomycota sp
OTU_1837	k__Fungi;p__Ascomycota;c__Eurotiomycetes;o__Chaetothyriales;f__Incertae sedis;g__Strelitziana;s__Strelitziana mali
OTU_1698	k__Fungi;p__Ascomycota;c__Dothideomycetes;o__Pleosporales;f__unidentified;g__unidentified;s__Pleosporales sp
OTU_152	k__Fungi;p__Ascomycota;c__Sordariomycetes;o__Trichosphaeriales;f__Incertae sedis;g__Nigrospora;s__Nigrospora sp FF_2011
OTU_153	k__Fungi;p__Ascomycota;c__Eurotiomycetes;o__Chaetothyriales;f__Chaetothyriaceae;g__unidentified;s__Chaetothyriaceae sp
OTU_154	k__Fungi;p__Ascomycota;c__Dothideomycetes;o__Capnodiales;f__Mycosphaerellaceae;g__Mycosphaerella;s__Mycosphaerella gregaria
OTU_155	k__Fungi;p__unidentified;c__unidentified;o__unidentified;f__unidentified;g__unidentified;s__Fungi sp
OTU_83	No blast hit
OTU_81	k__Fungi;p__Basidiomycota;c__Tremellomycetes;o__Tremellales;f__Incertae sedis;g__unidentified;s__Tremellales sp
OTU_175	k__Fungi;p__Basidiomycota;c__Microbotryomycetes;o__Sporidiobolales;f__Incertae sedis;g__Sporobolomyces;s__Sporobolomyces ogasawarensis
OTU_89	k__Fungi;p__Basidiomycota;c__Microbotryomycetes;o__Sporidiobolales;f__Incertae sedis;g__unidentified;s__Sporidiobolales sp
OTU_88	k__Fungi;p__Ascomycota;c__Incertae sedis;o__Incertae sedis;f__Incertae sedis;g__Zymoseptoria;s__Zymoseptoria ardabiliae
OTU_255	k__Fungi;p__Ascomycota;c__Eurotiomycetes;o__Chaetothyriales;f__Incertae sedis;g__Strelitziana;s__Strelitziana mali
OTU_313	k__Fungi;p__Ascomycota;c__Dothideomycetes;o__unidentified;f__unidentified;g__unidentified;s__Dothideomycetes sp
OTU_382	k__Fungi;p__Basidiomycota;c__Microbotryomycetes;o__Sporidiobolales;f__unidentified;g__unidentified;s__Sporidiobolales sp
OTU_380	k__Fungi;p__Basidiomycota;c__Tremellomycetes;o__Tremellales;f__Incertae sedis;g__Hannaella;s__Hannaella oryzae
OTU_389	k__Fungi;p__Ascomycota;c__Dothideomycetes;o__Pleosporales;f__Incertae sedis;g__Letendraea;s__Letendraea helminthicola
OTU_1535	k__Fungi;p__Ascomycota;c__unidentified;o__unidentified;f__unidentified;g__unidentified;s__Ascomycota sp
OTU_1331	k__Fungi;p__Ascomycota;c__Dothideomycetes;o__Pleosporales;f__Incertae sedis;g__Phoma;s__Phoma sp 6306
OTU_1713	k__Fungi;p__Ascomycota;c__Dothideomycetes;o__Pleosporales;f__Incertae sedis;g__Phoma;s__Phoma dactylidis
OTU_545	k__Fungi;p__Ascomycota;c__Eurotiomycetes;o__Chaetothyriales;f__Herpotrichiellaceae;g__Cladophialophora;s__Cladophialophora subtilis
OTU_146	k__Fungi;p__Basidiomycota;c__Tremellomycetes;o__Tremellales;f__unidentified;g__unidentified;s__Tremellales sp

	OTU_149	k__Fungi;p__Basidiomycota;c__unidentified;o__unidentified;f__unidentified;g__unidentified;s__Basidiomycota sp
	OTU_78	k__Fungi;p__Ascomycota;c__unidentified;o__unidentified;f__unidentified;g__unidentified;s__Ascomycota sp
	OTU_79	k__Fungi;p__Ascomycota;c__Dothideomycetes;o__Pleosporales;f__Incertae sedis;g__Boeremia;s__Boeremia exigua var. populi
	OTU_75	k__Fungi;p__Ascomycota;c__Eurotiomycetes;o__Chaetothyriales;f__Incertae sedis;g__Strelitziana;s__Strelitziana africana
	OTU_71	k__Fungi;p__Basidiomycota;c__Tremellomycetes;o__Tremellales;f__Incertae sedis;g__Dioszegia;s__Dioszegia catarinonii
Fungi	Raw Pu-erh	
	OTU_93	k__Fungi;p__Ascomycota;c__Dothideomycetes;o__Pleosporales;f__Pleosporaceae;g__Stemphylium;s__Stemphylium drummondii
	OTU_74	k__Fungi;p__Ascomycota;c__Saccharomycetes;o__Saccharomycetales;f__Saccharomycetaceae;g__Saccharomyces;s__Saccharomyces cerevisiae
	OTU_201	k__Fungi;p__Basidiomycota;c__Agaricomycetes;o__Boletales;f__Boletaceae;g__Boletus;s__Boletus edulis
	OTU_1462	k__Fungi;p__Ascomycota;c__Dothideomycetes;o__Myriangiales;f__unidentified;g__unidentified;s__Myriangiales sp
	OTU_35	k__Fungi;p__Ascomycota;c__Dothideomycetes;o__Capnodiales;f__Mycosphaerellaceae;g__Zasmidium;s__Zasmidium xenoparkii
	OTU_1518	k__Fungi;p__Ascomycota;c__Sordariomycetes;o__Sordariales;f__Sordariaceae;g__Neurospora;s__Neurospora terricola
	OTU_1915	k__Fungi;p__Ascomycota;c__Eurotiomycetes;o__Eurotiales;f__unidentified;g__unidentified;s__Eurotiales sp
	OTU_96	k__Fungi;p__Basidiomycota;c__Tremellomycetes;o__Cystofilobasidiales;f__Cystofilobasidiaceae;g__Cystofilobasidium;s__Cystofilobasidium infirmominiatum
	OTU_1690	k__Fungi;p__Ascomycota;c__Eurotiomycetes;o__Eurotiales;f__Trichocomaceae;g__Aspergillus;s__Aspergillus cibarius
	OTU_84	k__Fungi;p__Ascomycota;c__Dothideomycetes;o__Capnodiales;f__Davidiellaceae;g__Cladosporium;s__Cladosporium velox
Fungi	Ripened Pu-erh	
	OTU_1719	k__Fungi;p__Ascomycota;c__Eurotiomycetes;o__Eurotiales;f__Trichocomaceae;g__Aspergillus;s__Aspergillus piperis
	OTU_1486	k__Fungi;p__Ascomycota;c__Eurotiomycetes;o__Eurotiales;f__Trichocomaceae;g__unidentified;s__Trichocomaceae sp
	OTU_1895	k__Fungi;p__Ascomycota;c__Eurotiomycetes;o__Eurotiales;f__Trichocomaceae;g__unidentified;s__Trichocomaceae sp
	OTU_453	k__Fungi;p__Ascomycota;c__Eurotiomycetes;o__Eurotiales;f__Trichocomaceae;g__Aspergillus;s__Aspergillus niger
	OTU_1948	k__Fungi;p__Ascomycota;c__Eurotiomycetes;o__Eurotiales;f__Trichocomaceae;g__Aspergillus;s__Aspergillus niger
	OTU_41	k__Fungi;p__Zygomycota;c__Incertae sedis;o__Mucorales;f__Lichtheimiaceae;g__Rhizomucor;s__Rhizomucor pusillus
	OTU_1293	k__Fungi;p__Ascomycota;c__Eurotiomycetes;o__Eurotiales;f__Trichocomaceae;g__Aspergillus;s__Aspergillus niger
	OTU_3	k__Fungi;p__Ascomycota;c__Eurotiomycetes;o__Eurotiales;f__Trichocomaceae;g__Aspergillus;s__Aspergillus niger
	OTU_30	k__Fungi;p__Ascomycota;c__Eurotiomycetes;o__Eurotiales;f__Trichocomaceae;g__Penicillium;s__Penicillium brocae
	OTU_134	k__Fungi;p__Ascomycota;c__Dothideomycetes;o__Incertae sedis;f__Eremomycetaceae;g__Arthrographis;s__Arthrographis sp
	OTU_1788	k__Fungi;p__Ascomycota;c__Eurotiomycetes;o__Eurotiales;f__Trichocomaceae;g__Aspergillus;s__Aspergillus penicillioides
	OTU_368	k__Fungi;p__Ascomycota;c__Eurotiomycetes;o__Eurotiales;f__Incertae sedis;g__Thermomyces;s__Thermomyces lanuginosus
	OTU_263	k__Fungi;p__Ascomycota;c__Saccharomycetes;o__Saccharomycetales;f__unidentified;g__unidentified;s__Saccharomycetales sp
	OTU_1760	k__Fungi;p__Ascomycota;c__Eurotiomycetes;o__Eurotiales;f__Trichocomaceae;g__Aspergillus;s__Aspergillus niger
	OTU_1757	k__Fungi;p__Ascomycota;c__Eurotiomycetes;o__Eurotiales;f__Trichocomaceae;g__unidentified;s__Trichocomaceae sp
	OTU_10	k__Fungi;p__Ascomycota;c__Eurotiomycetes;o__Eurotiales;f__Trichocomaceae;g__unidentified;s__Trichocomaceae sp
	OTU_11	k__Fungi;p__Ascomycota;c__Eurotiomycetes;o__Eurotiales;f__Trichocomaceae;g__unidentified;s__Trichocomaceae sp
	OTU_221	k__Fungi;p__Ascomycota;c__Eurotiomycetes;o__Eurotiales;f__Trichocomaceae;g__Aspergillus;s__Aspergillus ochraceus
	OTU_381	k__Fungi;p__Ascomycota;c__Eurotiomycetes;o__Eurotiales;f__Trichocomaceae;g__Aspergillus;s__Aspergillus niger
	OTU_1555	k__Fungi;p__Ascomycota;c__Eurotiomycetes;o__Eurotiales;f__Trichocomaceae;g__Aspergillus;s__Aspergillus niger
Fungi	Raw+Ripened Pu-erh	
	OTU_1488	k__Fungi;p__Ascomycota;c__Eurotiomycetes;o__Eurotiales;f__Trichocomaceae;g__Aspergillus;s__Aspergillus restrictus
	OTU_1249	k__Fungi;p__Ascomycota;c__Eurotiomycetes;o__Eurotiales;f__Trichocomaceae;g__Aspergillus;s__Aspergillus penicillioides
	OTU_125	k__Fungi;p__Ascomycota;c__Eurotiomycetes;o__Eurotiales;f__Trichocomaceae;g__Aspergillus;s__Aspergillus penicillioides
	OTU_1714	k__Fungi;p__Ascomycota;c__Eurotiomycetes;o__Eurotiales;f__Trichocomaceae;g__Aspergillus;s__Aspergillus penicillioides
	OTU_1310	k__Fungi;p__Ascomycota;c__Eurotiomycetes;o__Eurotiales;f__Trichocomaceae;g__Aspergillus;s__Aspergillus niger
	OTU_1893	k__Fungi;p__Ascomycota;c__Eurotiomycetes;o__Eurotiales;f__unidentified;g__unidentified;s__Eurotiales sp
	OTU_1879	k__Fungi;p__Ascomycota;c__Eurotiomycetes;o__Eurotiales;f__Trichocomaceae;g__Aspergillus;s__Aspergillus penicillioides
	OTU_1652	k__Fungi;p__Ascomycota;c__Eurotiomycetes;o__Eurotiales;f__Trichocomaceae;g__Aspergillus;s__Aspergillus penicillioides
	OTU_1841	k__Fungi;p__Ascomycota;c__Eurotiomycetes;o__Eurotiales;f__Trichocomaceae;g__Aspergillus;s__Aspergillus penicillioides
	OTU_1562	k__Fungi;p__Ascomycota;c__Eurotiomycetes;o__Eurotiales;f__unidentified;g__unidentified;s__Eurotiales sp
	OTU_42	k__Fungi;p__Ascomycota;c__Eurotiomycetes;o__Eurotiales;f__Trichocomaceae;g__Aspergillus;s__Aspergillus sp DY115_21_7_M6
	OTU_1798	k__Fungi;p__Ascomycota;c__Eurotiomycetes;o__Eurotiales;f__unidentified;g__unidentified;s__Eurotiales sp
	OTU_1853	k__Fungi;p__Ascomycota;c__Eurotiomycetes;o__Eurotiales;f__Trichocomaceae;g__Aspergillus;s__Aspergillus penicillioides
	OTU_9	k__Fungi;p__Ascomycota;c__Dothideomycetes;o__Myriangiales;f__unidentified;g__unidentified;s__Myriangiales sp
	OTU_2	k__Fungi;p__Ascomycota;c__Eurotiomycetes;o__Eurotiales;f__unidentified;g__unidentified;s__Eurotiales sp
	OTU_1	k__Fungi;p__Ascomycota;c__Saccharomycetes;o__Saccharomycetales;f__Trichomonascaceae;g__Blastobotrys;s__Blastobotrys adeninivorans
	OTU_6	k__Fungi;p__Ascomycota;c__Eurotiomycetes;o__Eurotiales;f__Trichocomaceae;g__Penicillium;s__Penicillium citrinum
	OTU_4	k__Fungi;p__Ascomycota;c__Eurotiomycetes;o__Eurotiales;f__Incertae sedis;g__Thermomyces;s__Thermomyces lanuginosus
	OTU_1198	k__Fungi;p__Ascomycota;c__Eurotiomycetes;o__Eurotiales;f__Trichocomaceae;g__Aspergillus;s__Aspergillus penicillioides
	OTU_1619	k__Fungi;p__Ascomycota;c__Eurotiomycetes;o__Eurotiales;f__Trichocomaceae;g__Aspergillus;s__Aspergillus sp
	OTU_1199	k__Fungi;p__Ascomycota;c__Eurotiomycetes;o__Eurotiales;f__Trichocomaceae;g__Aspergillus;s__Aspergillus cibarius
	OTU_1825	k__Fungi;p__Ascomycota;c__Eurotiomycetes;o__Eurotiales;f__Trichocomaceae;g__Aspergillus;s__Aspergillus cibarius
	OTU_22	k__Fungi;p__Ascomycota;c__Sordariomycetes;o__Sordariales;f__Sordariaceae;g__Neurospora;s__Neurospora terricola
	OTU_27	k__Fungi;p__Ascomycota;c__Sordariomycetes;o__Hypocreales;f__Nectriaceae;g__Fusarium;s__Fusarium delphinoides
	OTU_1527	k__Fungi;p__Ascomycota;c__Eurotiomycetes;o__Eurotiales;f__Trichocomaceae;g__Aspergillus;s__Aspergillus penicillioides
	OTU_1059	k__Fungi;p__Ascomycota;c__Eurotiomycetes;o__Eurotiales;f__Trichocomaceae;g__Aspergillus;s__Aspergillus penicillioides
	OTU_1021	k__Fungi;p__Ascomycota;c__Dothideomycetes;o__Myriangiales;f__unidentified;g__unidentified;s__Myriangiales sp
	OTU_18	k__Fungi;p__Ascomycota;c__Eurotiomycetes;o__Eurotiales;f__Trichocomaceae;g__Aspergillus;s__Aspergillus subversicolor

	OTU_19	k__Fungi;p__Ascomycota;c__Eurotiomycetes;o__Eurotiales;f__Trichocomaceae;g__Aspergillus;s__Aspergillus penicillioides
	OTU_17	k__Fungi;p__Ascomycota;c__Sordariomycetes;o__Sordariales;f__Sordariaceae;g__Neurospora;s__Neurospora terricola
	OTU_12	k__Fungi;p__Ascomycota;c__Eurotiomycetes;o__Eurotiales;f__Trichocomaceae;g__Aspergillus;s__Aspergillus vitricola
	OTU_13	k__Fungi;p__Ascomycota;c__Eurotiomycetes;o__Eurotiales;f__Trichocomaceae;g__Aspergillus;s__Aspergillus restrictus
	OTU_1554	k__Fungi;p__Ascomycota;c__Eurotiomycetes;o__Eurotiales;f__Trichocomaceae;g__Aspergillus;s__Aspergillus penicillioides
	OTU_1626	k__Fungi;p__Ascomycota;c__Eurotiomycetes;o__Eurotiales;f__Trichocomaceae;g__Aspergillus;s__Aspergillus penicillioides
	OTU_1343	k__Fungi;p__Ascomycota;c__Eurotiomycetes;o__Eurotiales;f__unidentified;g__unidentified;s__Eurotiales sp
	OTU_1023	k__Fungi;p__Ascomycota;c__Eurotiomycetes;o__Eurotiales;f__unidentified;g__unidentified;s__Eurotiales sp
	OTU_1334	k__Fungi;p__Ascomycota;c__Eurotiomycetes;o__Eurotiales;f__unidentified;g__unidentified;s__Eurotiales sp
	OTU_1936	k__Fungi;p__Ascomycota;c__Eurotiomycetes;o__Eurotiales;f__Trichocomaceae;g__Aspergillus;s__Aspergillus penicillioides
	OTU_1727	k__Fungi;p__Ascomycota;c__Eurotiomycetes;o__Eurotiales;f__Trichocomaceae;g__Aspergillus;s__Aspergillus penicillioides
	OTU_1801	k__Fungi;p__Ascomycota;c__Saccharomycetes;o__Saccharomycetales;f__Trichomonascaceae;g__Blastobotrys;s__Blastobotrys adeninivorans
Bacteria	Fresh tea leaf	
	OTU_1811	k__Bacteria; p__Proteobacteria; c__Alphaproteobacteria; o__Sphingomonadales; f__Sphingomonadaceae; g__Sphingomonas; s__
	OTU_125	k__Bacteria; p__Proteobacteria; c__Alphaproteobacteria; o__Rhizobiales; f__Methylobacteriaceae; g__Methylobacterium; s__adhaesivum
	OTU_158	k__Bacteria; p__Proteobacteria; c__Alphaproteobacteria; o__Rhizobiales; f__Methylobacteriaceae; g__; s__
	OTU_65	k__Bacteria; p__Proteobacteria; c__Alphaproteobacteria; o__Rhizobiales; f__Methylobacteriaceae; g__Methylobacterium; s__
	OTU_64	k__Bacteria; p__Proteobacteria; c__Alphaproteobacteria; o__Sphingomonadales; f__Sphingomonadaceae; g__Sphingomonas; s__yabuuchiae
	OTU_426	k__Bacteria; p__Proteobacteria; c__Gammaproteobacteria; o__Pseudomonadales; f__Pseudomonadaceae; g__Pseudomonas; s__
	OTU_319	k__Bacteria; p__Proteobacteria; c__Gammaproteobacteria; o__Pseudomonadales; f__Pseudomonadaceae; g__Pseudomonas; s__viridiflava
	OTU_145	k__Bacteria; p__Actinobacteria; c__Actinobacteria; o__Actinomycetales; f__Microbacteriaceae; g__; s__
	OTU_456	k__Bacteria; p__Actinobacteria; c__Actinobacteria; o__Actinomycetales; f__Kineosporiaceae; g__Kineococcus; s__
	OTU_756	k__Bacteria; p__Actinobacteria; c__Actinobacteria; o__Actinomycetales; f__Microbacteriaceae; g__Curtobacterium; s__
	OTU_1474	k__Bacteria; p__Proteobacteria; c__Alphaproteobacteria; o__Rhizobiales; f__Methylobacteriaceae; g__; s__
	OTU_362	k__Bacteria; p__Proteobacteria; c__Alphaproteobacteria; o__Rhizobiales; f__Methylocystaceae; g__; s__
	OTU_1197	k__Bacteria; p__Proteobacteria; c__Alphaproteobacteria; o__Sphingomonadales; f__Sphingomonadaceae; g__; s__
	OTU_1081	k__Bacteria; p__Proteobacteria; c__Alphaproteobacteria; o__Rhizobiales; f__Methylobacteriaceae; g__Methylobacterium; s__organophilum
	OTU_21	k__Bacteria; p__Proteobacteria; c__Betaproteobacteria; o__Burkholderiales; f__; g__; s__
	OTU_569	k__Bacteria; p__Proteobacteria; c__Alphaproteobacteria; o__Sphingomonadales; f__Sphingomonadaceae; g__Sphingomonas; s__
	OTU_959	k__Bacteria; p__Cyanobacteria; c__Oscillatoriophycideae; o__Oscillatoriales; f__Phormidiaceae; g__; s__
	OTU_1238	k__Bacteria; p__Proteobacteria; c__Alphaproteobacteria; o__Rhizobiales; f__Rhizobiaceae; g__Agrobacterium; s__
	OTU_1331	k__Bacteria; p__Proteobacteria; c__Alphaproteobacteria; o__Sphingomonadales; f__Sphingomonadaceae; g__Sphingomonas; s__
	OTU_2461	k__Bacteria; p__Proteobacteria; c__Alphaproteobacteria; o__Sphingomonadales; f__Sphingomonadaceae; g__Sphingomonas; s__
	OTU_1502	k__Bacteria; p__Actinobacteria; c__Actinobacteria; o__Actinomycetales; f__Microbacteriaceae; g__Microbacterium; s__
	OTU_140	k__Bacteria; p__Proteobacteria; c__Gammaproteobacteria; o__Xanthomonadales; f__Xanthomonadaceae; g__Luteibacter; s__rhizovicinus
Bacteria	Raw Pu-erh	
	OTU_487	k__Bacteria; p__Actinobacteria; c__Actinobacteria; o__Actinomycetales; f__Micrococcaceae; g__Arthrobacter; s__
	OTU_246	k__Bacteria; p__Firmicutes; c__Clostridia; o__Clostridiales; f__Lachnospiraceae; g__Coprococcus; s__
	OTU_2143	k__Bacteria; p__Proteobacteria; c__Alphaproteobacteria; o__Rhizobiales; f__Methylobacteriaceae; g__Methylobacterium; s__adhaesivum
	OTU_170	k__Bacteria; p__[Thermi]; c__Deinococci; o__Deinococcales; f__Trueperaceae; g__Truepera; s__
	OTU_183	k__Bacteria; p__Firmicutes; c__Clostridia; o__Clostridiales; f__Peptostreptococcaceae; g__; s__
	OTU_945	k__Bacteria; p__Firmicutes; c__Clostridia; o__Clostridiales; f__Clostridiaceae; g__Clostridium; s__
	OTU_86	k__Bacteria; p__Firmicutes; c__Clostridia; o__Clostridiales; f__Clostridiaceae; g__; s__
	OTU_118	k__Bacteria; p__Firmicutes; c__Clostridia; o__Clostridiales; f__; g__; s__
	OTU_1683	k__Bacteria; p__Firmicutes; c__Bacilli; o__Bacillales; f__Planococcaceae; g__; s__
	OTU_1782	k__Bacteria; p__Actinobacteria; c__Actinobacteria; o__Actinomycetales; f__Pseudonocardiaceae; g__Saccharopolyspora; s__
	OTU_1102	k__Bacteria; p__Firmicutes; c__Bacilli; o__Turicibacterales; f__Turicibacteraceae; g__Turicibacter; s__
	OTU_264	k__Bacteria; p__Firmicutes; c__Clostridia; o__Clostridiales; f__Clostridiaceae; g__Clostridium; s__
	OTU_261	k__Bacteria; p__Chlamydiae; c__Chlamydiia; o__Chlamydiales; f__Parachlamydiaceae; g__Parachlamydia; s__
	OTU_303	k__Bacteria; p__Firmicutes; c__Bacilli; o__Bacillales; f__Bacillaceae; g__; s__
	OTU_124	k__Bacteria; p__Bacteroidetes; c__Sphingobacteriia; o__Sphingobacteriales; f__Sphingobacteriaceae; g__Pedobacter; s__
	OTU_491	k__Bacteria; p__Firmicutes; c__Clostridia; o__Clostridiales; f__Clostridiaceae; g__; s__
	OTU_250	k__Bacteria; p__Firmicutes; c__Clostridia; o__Clostridiales; f__Clostridiaceae; g__Clostridium; s__
	OTU_79	k__Bacteria; p__Firmicutes; c__Clostridia; o__Clostridiales; f__Peptostreptococcaceae; g__; s__
Bacteria	Ripened Pu-erh	
	OTU_321	k__Bacteria; p__Bacteroidetes; c__Sphingobacteriia; o__Sphingobacteriales; f__Sphingobacteriaceae; g__; s__
	OTU_249	k__Bacteria; p__Actinobacteria; c__Actinobacteria; o__Actinomycetales; f__; g__; s__
	OTU_393	k__Bacteria; p__Proteobacteria; c__Alphaproteobacteria; o__Rhodospirillales; f__Acetobacteraceae; g__; s__
	OTU_397	k__Bacteria; p__Firmicutes; c__Bacilli; o__Bacillales; f__Paenibacillaceae; g__Cohnella; s__
	OTU_436	k__Bacteria; p__Actinobacteria; c__Thermoleophilia; o__Solirubrobacterales; f__Patulibacteraceae; g__Patulibacter; s__
	OTU_438	k__Bacteria; p__Firmicutes; c__Clostridia; o__Clostridiales; f__Ruminococcaceae; g__; s__
	OTU_1437	k__Bacteria; p__Firmicutes; c__Bacilli; o__Bacillales; f__Staphylococcaceae; g__Staphylococcus; s__
	OTU_236	k__Bacteria; p__Firmicutes; c__Bacilli; o__Bacillales; f__Paenibacillaceae; g__Paenibacillus; s__hodogayensis
	OTU_1651	k__Bacteria; p__Proteobacteria; c__Alphaproteobacteria; o__Rhizobiales; f__Hyphomicrobiaceae; g__Devosia; s__
	OTU_1031	k__Bacteria; p__Firmicutes; c__Bacilli; o__Lactobacillales; f__Lactobacillaceae; g__Lactobacillus; s__
	OTU_2168	k__Bacteria; p__Bacteroidetes; c__Sphingobacteriia; o__Sphingobacteriales; f__Sphingobacteriaceae; g__; s__

OTU_1975	k__Bacteria; p__Actinobacteria; c__Actinobacteria; o__Actinomycetales; f__Dermabacteraceae; g__Brachybacterium; s__
OTU_2169	k__Bacteria; p__Actinobacteria; c__Actinobacteria; o__Actinomycetales; f__Micrococcaceae; g__; s__
OTU_2558	k__Bacteria; p__Proteobacteria; c__Betaproteobacteria; o__Burkholderiales; f__Alcaligenaceae; g__; s__
OTU_1521	k__Bacteria; p__Actinobacteria; c__Actinobacteria; o__Actinomycetales; f__Nocardioidaceae; g__; s__
OTU_1244	k__Bacteria; p__Firmicutes; c__Bacilli; o__Lactobacillales; f__Lactobacillaceae; g__Lactobacillus; s__
OTU_1085	k__Bacteria; p__Firmicutes; c__Bacilli; o__Bacillales; f__Bacillaceae; g__Bacillus; s__cohnii
OTU_1921	k__Bacteria; p__Actinobacteria; c__Actinobacteria; o__Actinomycetales; f__Micrococcaceae; g__; s__
OTU_1925	k__Bacteria; p__Firmicutes; c__Bacilli; o__Bacillales; f__Bacillaceae; g__Bacillus; s__
OTU_2262	k__Bacteria; p__Firmicutes; c__Bacilli; o__Bacillales; f__Sporolactobacillaceae; g__; s__
OTU_1866	k__Bacteria; p__Proteobacteria; c__Betaproteobacteria; o__Burkholderiales; f__Alcaligenaceae; g__Pigmentiphaga; s__
OTU_1867	k__Bacteria; p__Proteobacteria; c__Betaproteobacteria; o__Burkholderiales; f__Alcaligenaceae; g__; s__
OTU_2182	k__Bacteria; p__Actinobacteria; c__Actinobacteria; o__Actinomycetales; f__Microbacteriaceae; g__Leucobacter; s__
OTU_2445	k__Bacteria; p__Actinobacteria; c__Actinobacteria; o__Actinomycetales; f__Dermabacteraceae; g__Brachybacterium; s__
OTU_1468	k__Bacteria; p__Firmicutes; c__Bacilli; o__Lactobacillales; f__; g__; s__
OTU_1810	k__Bacteria; p__Proteobacteria; c__Betaproteobacteria; o__Rhodocyclales; f__Rhodocyclaceae; g__; s__
OTU_2544	k__Bacteria; p__Firmicutes; c__Bacilli; o__Lactobacillales; f__Lactobacillaceae; g__Pediococcus; s__
OTU_2064	k__Bacteria; p__Actinobacteria; c__Actinobacteria; o__Actinomycetales; f__Nocardioidaceae; g__Aeromicrobium; s__
OTU_1771	k__Bacteria; p__Firmicutes; c__Bacilli; o__Bacillales; f__Bacillaceae; g__Bacillus; s__
OTU_129	k__Bacteria; p__Proteobacteria; c__Alphaproteobacteria; o__Rhizobiales; f__Phyllobacteriaceae; g__; s__
OTU_127	k__Bacteria; p__Bacteroidetes; c__Flavobacteriia; o__Flavobacteriales; f__Flavobacteriaceae; g__; s__
OTU_120	k__Bacteria; p__Firmicutes; c__Bacilli; o__Bacillales; f__Paenibacillaceae; g__Ammoniphilus; s__
OTU_2358	k__Bacteria; p__Firmicutes; c__Bacilli; o__Bacillales; f__Planococcaceae; g__Staphylococcus; s__saprophyticus
OTU_69	k__Bacteria; p__Firmicutes; c__Bacilli; o__Bacillales; f__Paenibacillaceae; g__Paenibacillus; s__
OTU_156	k__Bacteria; p__Actinobacteria; c__Actinobacteria; o__Actinomycetales; f__Corynebacteriaceae; g__Corynebacterium; s__
OTU_60	k__Bacteria; p__Actinobacteria; c__Actinobacteria; o__Actinomycetales; f__Sanguibacteraceae; g__Sanguibacter; s__soli
OTU_63	k__Bacteria; p__Firmicutes; c__Bacilli; o__Bacillales; f__Bacillaceae; g__Bacillus; s__cereus
OTU_62	k__Bacteria; p__Actinobacteria; c__Actinobacteria; o__Actinomycetales; f__Actinopolysporaceae; g__; s__
OTU_883	k__Bacteria; p__Actinobacteria; c__Actinobacteria; o__Actinomycetales; f__Promicromonosporaceae; g__Cellulosimicrobium; s__
OTU_177	k__Bacteria; p__Firmicutes; c__Clostridia; o__Clostridiales; f__Lachnospiraceae; g__Coprococcus; s__
OTU_1401	k__Bacteria; p__Firmicutes; c__Bacilli; o__Bacillales; f__Planococcaceae; g__; s__
OTU_2416	k__Bacteria; p__Actinobacteria; c__Actinobacteria; o__Actinomycetales; f__Micrococcaceae; g__; s__
OTU_347	k__Bacteria; p__Proteobacteria; c__Deltaproteobacteria; o__Myxococcales; f__; g__; s__4e-134
OTU_421	k__Bacteria; p__Proteobacteria; c__Gammaproteobacteria; o__Enterobacteriales; f__Enterobacteriaceae; g__Escherichia; s__blattae
OTU_1460	k__Bacteria; p__Firmicutes; c__Bacilli; o__Bacillales; f__Bacillaceae; g__; s_5e-127
OTU_2258	k__Bacteria; p__Bacteroidetes; c__Sphingobacteriia; o__Sphingobacteriales; f__Sphingobacteriaceae; g__Sphingobacterium; s__
OTU_2509	k__Bacteria; p__Firmicutes; c__Bacilli; o__Bacillales; f__Bacillaceae; g__Bacillus; s__coagulans
OTU_317	k__Bacteria; p__Firmicutes; c__Bacilli; o__Bacillales; f__Bacillaceae; g__Bacillus; s_9e-132
OTU_1317	k__Bacteria; p__Actinobacteria; c__Actinobacteria; o__Actinomycetales; f__Brevibacteriaceae; g__Brevibacterium; s__
OTU_1442	k__Bacteria; p__Actinobacteria; c__Actinobacteria; o__Actinomycetales; f__Microbacteriaceae; g__Pseudoclavibacter; s__bifida
OTU_768	k__Bacteria; p__Firmicutes; c__Bacilli; o__Bacillales; f__Paenibacillaceae; g__Paenibacillus; s__
OTU_1894	k__Bacteria; p__Firmicutes; c__Bacilli; o__Bacillales; f__Paenibacillaceae; g__Paenibacillus; s__
OTU_1869	k__Bacteria; p__Firmicutes; c__Bacilli; o__Bacillales; f__; g__; s__
OTU_2270	k__Bacteria; p__Actinobacteria; c__Actinobacteria; o__Actinomycetales; f__Brevibacteriaceae; g__Brevibacterium; s__aureum
OTU_2273	k__Bacteria; p__Bacteroidetes; c__Sphingobacteriia; o__Sphingobacteriales; f__Sphingobacteriaceae; g__Sphingobacterium; s__multivorum
OTU_2154	k__Bacteria; p__Actinobacteria; c__Actinobacteria; o__Actinomycetales; f__Micrococcaceae; g__; s__
OTU_2151	k__Bacteria; p__Firmicutes; c__Bacilli; o__Bacillales; f__Staphylococcaceae; g__Staphylococcus; s__
OTU_2153	k__Bacteria; p__Actinobacteria; c__Actinobacteria; o__Actinomycetales; f__Micrococcaceae; g__; s__
OTU_59	k__Bacteria; p__Actinobacteria; c__Actinobacteria; o__Actinomycetales; f__Micrococcaceae; g__Arthrobacter; s__
OTU_50	k__Bacteria; p__Bacteroidetes; c__Sphingobacteriia; o__Sphingobacteriales; f__Sphingobacteriaceae; g__Sphingobacterium; s__
OTU_51	k__Bacteria; p__Bacteroidetes; c__Sphingobacteriia; o__Sphingobacteriales; f__Sphingobacteriaceae; g__; s__
OTU_52	k__Bacteria; p__Actinobacteria; c__Actinobacteria; o__Actinomycetales; f__Pseudonocardiaceae; g__Saccharopolyspora; s__hirsuta
OTU_53	k__Bacteria; p__Actinobacteria; c__Actinobacteria; o__Actinomycetales; f__Micrococcaceae; g__; s__
OTU_54	k__Bacteria; p__Firmicutes; c__Bacilli; o__Bacillales; f__Thermoactinomycetaceae; g__; s__
OTU_55	k__Bacteria; p__Firmicutes; c__Bacilli; o__Bacillales; f__Sporolactobacillaceae; g__Tuberibacillus; s__calidus
OTU_57	k__Bacteria; p__Actinobacteria; c__Actinobacteria; o__Actinomycetales; f__Brevibacteriaceae; g__Brevibacterium; s__
OTU_229	k__Bacteria; p__Firmicutes; c__Clostridia; o__Clostridiales; f__Peptococcaceae; g__Desulfosporosinus; s__meridiei
OTU_184	k__Bacteria; p__Actinobacteria; c__Actinobacteria; o__Actinomycetales; f__Microbacteriaceae; g__; s__
OTU_94	k__Bacteria; p__Proteobacteria; c__Alphaproteobacteria; o__Rhizobiales; f__Beijerinckiaceae; g__; s__
OTU_2462	k__Bacteria; p__Actinobacteria; c__Actinobacteria; o__Actinomycetales; f__Nocardioidaceae; g__; s__
OTU_162	k__Bacteria; p__Firmicutes; c__Bacilli; o__Bacillales; f__Paenibacillaceae; g__Paenibacillus; s__
OTU_2136	k__Bacteria; p__Actinobacteria; c__Actinobacteria; o__Actinomycetales; f__Brevibacteriaceae; g__Brevibacterium; s__
OTU_97	k__Bacteria; p__Firmicutes; c__Bacilli; o__Bacillales; f__Paenibacillaceae; g__Brevibacillus; s__reuszeri
OTU_91	k__Bacteria; p__Firmicutes; c__Clostridia; o__Clostridiales; f__Lachnospiraceae; g__Coprococcus; s__
OTU_143	k__Bacteria; p__Bacteroidetes; c__Sphingobacteriia; o__Sphingobacteriales; f__Sphingobacteriaceae; g__; s__
OTU_821	k__Bacteria; p__Firmicutes; c__Bacilli; o__Bacillales; f__Paenibacillaceae; g__Paenibacillus; s__
OTU_350	k__Bacteria; p__Firmicutes; c__Bacilli; o__Bacillales; f__Bacillaceae; g__Bacillus; s_4e-134

OTU_457	k__Bacteria; p__Firmicutes; c__Bacilli; o__Bacillales; f__Paenibacillaceae; g__Cohnella; s__
OTU_1569	k__Bacteria; p__Firmicutes; c__Bacilli; o__Bacillales; f__Bacillaceae; g__Bacillus; s__thermoamylovorans
OTU_142	k__Bacteria; p__Firmicutes; c__Bacilli; o__Bacillales; f__Paenibacillaceae; g__Paenibacillus; s__
OTU_98	k__Bacteria; p__Actinobacteria; c__Actinobacteria; o__Actinomycetales; f__Gordoniaceae; g__Gordonia; s__
OTU_2650	k__Bacteria; p__Firmicutes; c__Bacilli; o__Bacillales; f__Staphylococcaceae; g__Staphylococcus; s__succinus
OTU_799	k__Bacteria; p__Actinobacteria; c__Actinobacteria; o__Actinomycetales; f__Nocardioidaceae; g__Aeromicrobium; s__
OTU_1172	k__Bacteria; p__Proteobacteria; c__Alphaproteobacteria; o__Rhizobiales; f__Brucellaceae; g__; s__
OTU_856	k__Bacteria; p__Actinobacteria; c__Actinobacteria; o__Actinomycetales; f__Micrococcaceae; g__; s__
OTU_2488	k__Bacteria; p__Proteobacteria; c__Alphaproteobacteria; o__Rhizobiales; f__Brucellaceae; g__Ochrobactrum; s__
OTU_2655	k__Bacteria; p__Actinobacteria; c__Actinobacteria; o__Actinomycetales; f__Brevibacteriaceae; g__Brevibacterium; s__aureum
OTU_2526	k__Bacteria; p__Actinobacteria; c__Actinobacteria; o__Actinomycetales; f__Brevibacteriaceae; g__Brevibacterium; s__aureum
OTU_1265	k__Bacteria; p__Actinobacteria; c__Actinobacteria; o__Actinomycetales; f__Streptomycetaceae; g__; s__
OTU_269	k__Bacteria; p__Firmicutes; c__Clostridia; o__Clostridiales; f__Ruminococcaceae; g__Ruminococcus; s__
OTU_187	k__Bacteria; p__Actinobacteria; c__Actinobacteria; o__Actinomycetales; f__Dietziaceae; g__Dietzia; s__timorensis
OTU_2137	k__Bacteria; p__Proteobacteria; c__Alphaproteobacteria; o__Rhodobacterales; f__Rhodobacteraceae; g__Paracoccus; s__
OTU_2392	k__Bacteria; p__Firmicutes; c__Bacilli; o__Bacillales; f__Bacillaceae; g__Bacillus; s__coagulans
OTU_1844	k__Bacteria; p__Firmicutes; c__Bacilli; o__Bacillales; f__Bacillaceae; g__Bacillus; s__coagulans
OTU_1563	k__Bacteria; p__Firmicutes; c__Bacilli; o__Bacillales; f__Bacillaceae; g__Bacillus; s__coagulans
OTU_1841	k__Bacteria; p__Actinobacteria; c__Actinobacteria; o__Actinomycetales; f__Streptomycetaceae; g__; s__
OTU_2466	k__Bacteria; p__Firmicutes; c__Bacilli; o__Bacillales; f__; g__; s__
OTU_2463	k__Bacteria; p__Firmicutes; c__Bacilli; o__Bacillales; f__Bacillaceae; g__Bacillus; s__coagulans
OTU_2261	k__Bacteria; p__Actinobacteria; c__Actinobacteria; o__Actinomycetales; f__Brevibacteriaceae; g__Brevibacterium; s__
OTU_1281	k__Bacteria; p__Actinobacteria; c__Actinobacteria; o__Actinomycetales; f__Nocardioidaceae; g__Aeromicrobium; s__
OTU_121	k__Bacteria; p__Bacteroidetes; c__Sphingobacteriia; o__Sphingobacteriales; f__Sphingobacteriaceae; g__; s__
OTU_1283	k__Bacteria; p__Firmicutes; c__Bacilli; o__Bacillales; f__Bacillaceae; g__Bacillus; s__thermoamylovorans
OTU_1287	k__Bacteria; p__Firmicutes; c__Bacilli; o__Bacillales; f__; g__; s__
OTU_2522	k__Bacteria; p__Firmicutes; c__Bacilli; o__Lactobacillales; f__; g__; s__
OTU_2483	k__Bacteria; p__Firmicutes; c__Bacilli; o__Bacillales; f__Paenibacillaceae; g__Paenibacillus; s__
OTU_2001	k__Bacteria; p__Actinobacteria; c__Actinobacteria; o__Actinomycetales; f__Micrococcaceae; g__Sinomonas; s__
OTU_101	k__Bacteria; p__Bacteroidetes; c__Cytophagia; o__Cytophagales; f__Cytophagaceae; g__; s__
OTU_103	k__Bacteria; p__Proteobacteria; c__Gammaproteobacteria; o__Xanthomonadales; f__Xanthomonadaceae; g__Stenotrophomonas; s__acidaminiphila
OTU_105	k__Bacteria; p__Proteobacteria; c__Gammaproteobacteria; o__Xanthomonadales; f__Xanthomonadaceae; g__Pseudoxanthomonas; s__taiwanensis
OTU_1646	k__Bacteria; p__Firmicutes; c__Bacilli; o__Bacillales; f__Paenibacillaceae; g__Brevibacillus; s__
OTU_2584	k__Bacteria; p__Actinobacteria; c__Actinobacteria; o__Actinomycetales; f__Micrococcaceae; g__; s__
OTU_46	k__Bacteria; p__Firmicutes; c__Clostridia; o__Clostridiales; f__Lachnospiraceae; g__Coprococcus; s__
OTU_45	k__Bacteria; p__Firmicutes; c__Bacilli; o__Bacillales; f__Planococcaceae; g__; s__
OTU_42	k__Bacteria; p__Actinobacteria; c__Actinobacteria; o__Actinomycetales; f__Jonesiaceae; g__; s__
OTU_41	k__Bacteria; p__Firmicutes; c__Bacilli; o__Lactobacillales; f__Lactobacillaceae; g__Lactobacillus; s__acidipiscis
OTU_1872	k__Bacteria; p__Firmicutes; c__Bacilli; o__Bacillales; f__Sporolactobacillaceae; g__Tuberibacillus; s__calidus
OTU_1871	k__Bacteria; p__Firmicutes; c__Bacilli; o__Lactobacillales; f__Enterococcaceae; g__Enterococcus; s__
OTU_210	k__Bacteria; p__Bacteroidetes; c__[Saprospirae]; o__[Saprospirales]; f__Chitinophagaceae; g__; s__
OTU_586	k__Bacteria; p__Firmicutes; c__Bacilli; o__Bacillales; f__Bacillaceae; g__; s__
OTU_198	k__Bacteria; p__Firmicutes; c__Clostridia; o__Clostridiales; f__Lachnospiraceae; g__Coprococcus; s__
OTU_996	k__Bacteria; p__Actinobacteria; c__Actinobacteria; o__Actinomycetales; f__Brevibacteriaceae; g__Brevibacterium; s__
OTU_2453	k__Bacteria; p__Firmicutes; c__Bacilli; o__Bacillales; f__Bacillaceae; g__Bacillus; s__
OTU_2482	k__Bacteria; p__Firmicutes; c__Bacilli; o__Lactobacillales; f__Lactobacillaceae; g__Lactobacillus; s__
OTU_113	k__Bacteria; p__Firmicutes; c__Bacilli; o__Bacillales; f__Paenibacillaceae; g__Cohnella; s__
OTU_1743	k__Bacteria; p__Firmicutes; c__Bacilli; o__Bacillales; f__Staphylococcaceae; g__Staphylococcus; s__sciuri
OTU_964	k__Bacteria; p__Firmicutes; c__Bacilli; o__Bacillales; f__Paenibacillaceae; g__Paenibacillus; s__
OTU_1748	k__Bacteria; p__Actinobacteria; c__Actinobacteria; o__Actinomycetales; f__Micrococcaceae; g__; s__
OTU_119	k__Bacteria; p__Actinobacteria; c__Actinobacteria; o__Actinomycetales; f__Microbacteriaceae; g__Leucobacter; s__
OTU_2286	k__Bacteria; p__Actinobacteria; c__Actinobacteria; o__Actinomycetales; f__Actinomycetaceae; g__Bogoriella; s__caseolytica
OTU_2220	k__Bacteria; p__Actinobacteria; c__Actinobacteria; o__Actinomycetales; f__Brevibacteriaceae; g__Brevibacterium; s__aureum
OTU_1376	k__Bacteria; p__Actinobacteria; c__Actinobacteria; o__Actinomycetales; f__Micrococcaceae; g__; s__
OTU_2118	k__Bacteria; p__Firmicutes; c__Bacilli; o__Lactobacillales; f__Enterococcaceae; g__Enterococcus; s__
OTU_2551	k__Bacteria; p__Firmicutes; c__Bacilli; o__Bacillales; f__Bacillaceae; g__Bacillus; s__coagulans
OTU_2552	k__Bacteria; p__Firmicutes; c__Bacilli; o__Lactobacillales; f__Enterococcaceae; g__Enterococcus; s__haemoperoxidus
OTU_1206	k__Bacteria; p__Proteobacteria; c__Gammaproteobacteria; o__Pseudomonadales; f__Pseudomonadaceae; g__Pseudomonas; s__
OTU_1766	k__Bacteria; p__Actinobacteria; c__Actinobacteria; o__Actinomycetales; f__Ruaniaceae; g__; s__
OTU_1567	k__Bacteria; p__Proteobacteria; c__Betaproteobacteria; o__Burkholderiales; f__Oxalobacteraceae; g__; s__
OTU_2491	k__Bacteria; p__Proteobacteria; c__Betaproteobacteria; o__Burkholderiales; f__Alcaligenaceae; g__Achromobacter; s__
OTU_2648	k__Bacteria; p__Actinobacteria; c__Actinobacteria; o__Actinomycetales; f__Brevibacteriaceae; g__Brevibacterium; s__aureum
OTU_1851	k__Bacteria; p__Firmicutes; c__Bacilli; o__Bacillales; f__Bacillaceae; g__Bacillus; s__coagulans
OTU_1852	k__Bacteria; p__Actinobacteria; c__Actinobacteria; o__Actinomycetales; f__Micrococcaceae; g__Sinomonas; s__
OTU_1857	k__Bacteria; p__Firmicutes; c__Bacilli; o__Bacillales; f__Planococcaceae; g__; s__
OTU_1993	k__Bacteria; p__Firmicutes; c__Bacilli; o__Bacillales; f__Bacillaceae; g__Bacillus; s__cohnii

OTU_1996	k__Bacteria; p__Firmicutes; c__Clostridia; o__Clostridiales; f__Lachnospiraceae; g__Coprococcus; s__
OTU_2086	k__Bacteria; p__Actinobacteria; c__Actinobacteria; o__Actinomycetales; f__Brevibacteriaceae; g__Brevibacterium; s__aureum
OTU_2130	k__Bacteria; p__Actinobacteria; c__Actinobacteria; o__Actinomycetales; f__Microbacteriaceae; g__Leucobacter; s__
OTU_2256	k__Bacteria; p__Actinobacteria; c__Actinobacteria; o__Actinomycetales; f__Dermabacteraceae; g__Brachybacterium; s__
OTU_2252	k__Bacteria; p__Proteobacteria; c__Betaproteobacteria; o__Burkholderiales; f__Alcaligenaceae; g__Pigmentiphaga; s__
OTU_1296	k__Bacteria; p__Firmicutes; c__Bacilli; o__Bacillales; f__Paenibacillaceae; g__Paenibacillus; s__
OTU_2537	k__Bacteria; p__Firmicutes; c__Bacilli; o__Bacillales; f__Bacillaceae; g__Oceanobacillus; s__caeni
OTU_6	k__Bacteria; p__Firmicutes; c__Bacilli; o__Bacillales; f__Bacillaceae; g__Bacillus; s__coagulans
OTU_5	k__Bacteria; p__Firmicutes; c__Bacilli; o__Bacillales; f__Staphylococcaceae; g__Staphylococcus; s__succinus
OTU_2033	k__Bacteria; p__Firmicutes; c__Bacilli; o__Bacillales; f__Bacillaceae; g__Bacillus; s__coagulans
OTU_32	k__Bacteria; p__Actinobacteria; c__Actinobacteria; o__Actinomycetales; f__Corynebacteriaceae; g__Corynebacterium; s__variabile
OTU_30	k__Bacteria; p__Actinobacteria; c__Actinobacteria; o__Actinomycetales; f__Streptomycetaceae; g__; s__
OTU_31	k__Bacteria; p__Proteobacteria; c__Alphaproteobacteria; o__Rhizobiales; f__Brucellaceae; g__Ochrobactrum; s__
OTU_37	k__Bacteria; p__Firmicutes; c__Bacilli; o__Bacillales; f__Paenibacillaceae; g__Paenibacillus; s__
OTU_35	k__Bacteria; p__Actinobacteria; c__Actinobacteria; o__Actinomycetales; f__Nocardioidaceae; g__Aeromicrobium; s__
OTU_38	k__Bacteria; p__Proteobacteria; c__Betaproteobacteria; o__Burkholderiales; f__Alcaligenaceae; g__Achromobacter; s__
OTU_39	k__Bacteria; p__Actinobacteria; c__Actinobacteria; o__Actinomycetales; f__Brevibacteriaceae; g__Brevibacterium; s__
OTU_2109	k__Bacteria; p__Actinobacteria; c__Actinobacteria; o__Actinomycetales; f__Microbacteriaceae; g__Leucobacter; s__
OTU_581	k__Bacteria; p__Actinobacteria; c__Actinobacteria; o__Actinomycetales; f__Microbacteriaceae; g__Leucobacter; s__
OTU_2105	k__Bacteria; p__Actinobacteria; c__Actinobacteria; o__Actinomycetales; f__Microbacteriaceae; g__Leucobacter; s__
OTU_202	k__Bacteria; p__Firmicutes; c__Bacilli; o__Bacillales; f__Bacillaceae; g__; s__
OTU_200	k__Bacteria; p__Firmicutes; c__Bacilli; o__Bacillales; f__Paenibacillaceae; g__Paenibacillus; s__
OTU_204	k__Bacteria; p__Actinobacteria; c__Actinobacteria; o__Actinomycetales; f__Nocardiopsaceae; g__Thermobifida; s__alba
OTU_209	k__Bacteria; p__Firmicutes; c__Bacilli; o__Bacillales; f__Paenibacillaceae; g__Paenibacillus; s__
OTU_1470	k__Bacteria; p__Bacteroidetes; c__Sphingobacteriia; o__Sphingobacteriales; f__Sphingobacteriaceae; g__Sphingobacterium; s__
OTU_110	k__Bacteria; p__Bacteroidetes; c__Sphingobacteriia; o__Sphingobacteriales; f__Sphingobacteriaceae; g__Sphingobacterium; s__
OTU_111	k__Bacteria; p__Bacteroidetes; c__Sphingobacteriia; o__Sphingobacteriales; f__Sphingobacteriaceae; g__; s__
OTU_294	k__Bacteria; p__Firmicutes; c__Clostridia; o__Clostridiales; f__Lachnospiraceae; g__; s__
OTU_272	k__Bacteria; p__Firmicutes; c__Clostridia; o__Clostridiales; f__[Tissierellaceae]; g__Sedimentibacter; s__
OTU_374	k__Bacteria; p__Firmicutes; c__Bacilli; o__Bacillales; f__Bacillaceae; g__; s__
OTU_372	k__Bacteria; p__Actinobacteria; c__Actinobacteria; o__Actinomycetales; f__Nocardioidaceae; g__; s__
OTU_279	No blast hit
OTU_686	k__Bacteria; p__Actinobacteria; c__Actinobacteria; o__Actinomycetales; f__Microbacteriaceae; g__; s__
OTU_138	k__Bacteria; p__Firmicutes; c__Bacilli; o__Bacillales; f__Paenibacillaceae; g__Paenibacillus; s__
OTU_872	k__Bacteria; p__Firmicutes; c__Bacilli; o__Bacillales; f__Paenibacillaceae; g__Cohnella; s__
OTU_135	k__Bacteria; p__Firmicutes; c__Bacilli; o__Bacillales; f__Paenibacillaceae; g__Paenibacillus; s__ginsengihumi
OTU_2428	k__Bacteria; p__Actinobacteria; c__Actinobacteria; o__Actinomycetales; f__Micrococcaceae; g__; s__
OTU_137	k__Bacteria; p__Firmicutes; c__Bacilli; o__Bacillales; f__Paenibacillaceae; g__Paenibacillus; s__
OTU_130	k__Bacteria; p__Bacteroidetes; c__[Saprospirae]; o__[Saprospirales]; f__Chitinophagaceae; g__; s__
OTU_1613	k__Bacteria; p__Proteobacteria; c__Betaproteobacteria; o__Burkholderiales; f__Alcaligenaceae; g__; s__
OTU_1107	k__Bacteria; p__Proteobacteria; c__Betaproteobacteria; o__Rhodocyclales; f__Rhodocyclaceae; g__; s__
OTU_1104	k__Bacteria; p__Firmicutes; c__Bacilli; o__Lactobacillales; f__Lactobacillaceae; g__Pediococcus; s__
OTU_190	k__Bacteria; p__Proteobacteria; c__Deltaproteobacteria; o__Myxococcales; f__Myxococcaceae; g__Myxococcus; s__
OTU_2631	k__Bacteria; p__Bacteroidetes; c__Sphingobacteriia; o__Sphingobacteriales; f__Sphingobacteriaceae; g__; s__
OTU_1044	k__Bacteria; p__Firmicutes; c__Bacilli; o__Bacillales; f__Sporolactobacillaceae; g__; s__
OTU_361	k__Bacteria; p__Proteobacteria; c__Alphaproteobacteria; o__Rhizobiales; f__Hyphomicrobiaceae; g__Devosia; s__
OTU_1969	k__Bacteria; p__Firmicutes; c__Bacilli; o__Bacillales; f__Sporolactobacillaceae; g__; s__
OTU_2280	k__Bacteria; p__Actinobacteria; c__Actinobacteria; o__Actinomycetales; f__Brevibacteriaceae; g__Brevibacterium; s__aureum
OTU_2369	k__Bacteria; p__Firmicutes; c__Bacilli; o__Bacillales; f__Bacillaceae; g__Bacillus; s__coagulans
OTU_1820	k__Bacteria; p__Actinobacteria; c__Actinobacteria; o__Actinomycetales; f__Micrococcaceae; g__; s__
OTU_1910	k__Bacteria; p__Actinobacteria; c__Actinobacteria; o__Actinomycetales; f__Rarobacteraceae; g__Rarobacter; s__
OTU_1911	k__Bacteria; p__Firmicutes; c__Bacilli; o__Lactobacillales; f__Lactobacillaceae; g__Pediococcus; s__
OTU_1786	k__Bacteria; p__Actinobacteria; c__Actinobacteria; o__Actinomycetales; f__Dermabacteraceae; g__Brachybacterium; s__
OTU_2123	k__Bacteria; p__Actinobacteria; c__Actinobacteria; o__Actinomycetales; f__Dermabacteraceae; g__Brachybacterium; s__
OTU_2026	k__Bacteria; p__Actinobacteria; c__Actinobacteria; o__Actinomycetales; f__Actinomycetaceae; g__Bogoriella; s__caseolytica
OTU_2022	k__Bacteria; p__Firmicutes; c__Bacilli; o__Bacillales; f__Bacillaceae; g__Bacillus; s__thermoamylovorans
OTU_2433	k__Bacteria; p__Firmicutes; c__Bacilli; o__Lactobacillales; f__Lactobacillaceae; g__; s__
OTU_2225	k__Bacteria; p__Firmicutes; c__Bacilli; o__Bacillales; f__Bacillaceae; g__Bacillus; s__
OTU_1796	k__Bacteria; p__Bacteroidetes; c__Sphingobacteriia; o__Sphingobacteriales; f__Sphingobacteriaceae; g__Sphingobacterium; s__multivorum
OTU_2330	k__Bacteria; p__Actinobacteria; c__Actinobacteria; o__Actinomycetales; f__Pseudonocardiaceae; g__Saccharopolyspora; s__
OTU_20	k__Bacteria; p__Firmicutes; c__Bacilli; o__Bacillales; f__Listeriaceae; g__Listeria; s__grayi
OTU_27	k__Bacteria; p__Actinobacteria; c__Actinobacteria; o__Actinomycetales; f__Microbacteriaceae; g__Leucobacter; s__
OTU_26	k__Bacteria; p__Proteobacteria; c__Gammaproteobacteria; o__Pseudomonadales; f__Pseudomonadaceae; g__Pseudomonas; s__
OTU_29	k__Bacteria; p__Proteobacteria; c__Betaproteobacteria; o__Burkholderiales; f__Alcaligenaceae; g__Rhodospirillum; s__rubrum
OTU_1792	k__Bacteria; p__Actinobacteria; c__Actinobacteria; o__Actinomycetales; f__Micrococcaceae; g__; s__
OTU_2058	k__Bacteria; p__Firmicutes; c__Bacilli; o__Bacillales; f__Bacillaceae; g__Bacillus; s__

OTU_2052	k__Bacteria; p__Actinobacteria; c__Actinobacteria; o__Actinomycetales; f__Dermabacteraceae; g__Brachybacterium; s__
OTU_2608	k__Bacteria; p__Actinobacteria; c__Actinobacteria; o__Actinomycetales; f__Brevibacteriaceae; g__Brevibacterium; s__
OTU_564	k__Bacteria; p__Firmicutes; c__Bacilli; o__Lactobacillales; f__Lactobacillaceae; g__Lactobacillus; s__brevis
OTU_181	k__Bacteria; p__Firmicutes; c__Bacilli; o__Bacillales; f__Paenibacillaceae; g__Paenibacillus; s__
OTU_96	k__Bacteria; p__Firmicutes; c__Bacilli; o__Bacillales; f__; g__; s__
OTU_165	k__Bacteria; p__Proteobacteria; c__Betaproteobacteria; o__Methylophilales; f__Methylophilaceae; g__; s__
OTU_169	k__Bacteria; p__Actinobacteria; c__Actinobacteria; o__Actinomycetales; f__Pseudonocardiaceae; g__Prauserella; s__rugosa
OTU_286	k__Bacteria; p__Firmicutes; c__Bacilli; o__Bacillales; f__Paenibacillaceae; g__; s__
OTU_464	k__Bacteria; p__Verrucomicrobia; c__[Spartobacteria]; o__[Chthoniobacterales]; f__[Chthoniobacteraceae]; g__heteroC45_4W; s__
OTU_1594	k__Bacteria; p__Firmicutes; c__Bacilli; o__Bacillales; f__Bacillaceae; g__Halalkalibacillus; s__halophilus
OTU_223	k__Bacteria; p__Firmicutes; c__Bacilli; o__Lactobacillales; f__; g__; s__
OTU_225	k__Bacteria; p__Firmicutes; c__Clostridia; o__Clostridiales; f__Clostridiaceae; g__Alkaliphilus; s__transvaalensis
OTU_227	k__Bacteria; p__Firmicutes; c__AHT28; o__; f__; g__; s__
OTU_415	k__Bacteria; p__Actinobacteria; c__Actinobacteria; o__Actinomycetales; f__Streptomycetaceae; g__Streptomyces; s__
OTU_419	k__Bacteria; p__Proteobacteria; c__Alphaproteobacteria; o__Rhodobacterales; f__Rhodobacteraceae; g__Paracoccus; s__aminovorans
OTU_418	k__Bacteria; p__Actinobacteria; c__Actinobacteria; o__Actinomycetales; f__Brevibacteriaceae; g__Brevibacterium; s__
OTU_865	k__Bacteria; p__Firmicutes; c__Bacilli; o__Bacillales; f__Paenibacillaceae; g__Paenibacillus; s__
OTU_550	k__Bacteria; p__Actinobacteria; c__Actinobacteria; o__Actinomycetales; f__Promicromonosporaceae; g__Luteimicrobium; s__subarcticum
OTU_1133	k__Bacteria; p__Firmicutes; c__Bacilli; o__Bacillales; f__Paenibacillaceae; g__Paenibacillus; s__
OTU_1131	k__Bacteria; p__Actinobacteria; c__Actinobacteria; o__Actinomycetales; f__Brevibacteriaceae; g__Brevibacterium; s__
OTU_1355	k__Bacteria; p__Firmicutes; c__Bacilli; o__Bacillales; f__Staphylococcaceae; g__Staphylococcus; s__sciuri
OTU_1050	k__Bacteria; p__Actinobacteria; c__Actinobacteria; o__Actinomycetales; f__Promicromonosporaceae; g__Xylanimicrobium; s__
OTU_1799	k__Bacteria; p__Actinobacteria; c__Actinobacteria; o__Actinomycetales; f__Brevibacteriaceae; g__Brevibacterium; s__aureum
OTU_1764	k__Bacteria; p__Firmicutes; c__Bacilli; o__Bacillales; f__Paenibacillaceae; g__Paenibacillus; s__
OTU_2590	k__Bacteria; p__Actinobacteria; c__Actinobacteria; o__Actinomycetales; f__Dermabacteraceae; g__Brachybacterium; s__
OTU_1809	k__Bacteria; p__Firmicutes; c__Bacilli; o__Bacillales; f__Planococcaceae; g__; s__
OTU_2292	k__Bacteria; p__Actinobacteria; c__Actinobacteria; o__Actinomycetales; f__Pseudonocardiaceae; g__Saccharopolyspora; s__
OTU_2351	k__Bacteria; p__Actinobacteria; c__Actinobacteria; o__Actinomycetales; f__Dermabacteraceae; g__Brachybacterium; s__
OTU_2006	k__Bacteria; p__Firmicutes; c__Bacilli; o__Bacillales; f__Bacillaceae; g__Bacillus; s__coagulans
OTU_2331	k__Bacteria; p__Firmicutes; c__Bacilli; o__Bacillales; f__Bacillaceae; g__Bacillus; s__coagulans
OTU_1909	k__Bacteria; p__Firmicutes; c__Bacilli; o__Bacillales; f__Paenibacillaceae; g__Paenibacillus; s__
OTU_1908	k__Bacteria; p__Actinobacteria; c__Actinobacteria; o__Actinomycetales; f__Dermabacteraceae; g__Brachybacterium; s__
OTU_1422	k__Bacteria; p__Firmicutes; c__Bacilli; o__Bacillales; f__Sporolactobacillaceae; g__; s__
OTU_2193	k__Bacteria; p__Firmicutes; c__Bacilli; o__Bacillales; f__Planococcaceae; g__Staphylococcus; s__saprophyticus
OTU_2580	k__Bacteria; p__Actinobacteria; c__Actinobacteria; o__Actinomycetales; f__Nocardioidaceae; g__; s__
OTU_2586	k__Bacteria; p__Actinobacteria; c__Actinobacteria; o__Actinomycetales; f__Dermabacteraceae; g__Brachybacterium; s__
OTU_2427	k__Bacteria; p__Actinobacteria; c__Actinobacteria; o__Actinomycetales; f__Micrococcaceae; g__; s__
OTU_2342	k__Bacteria; p__Firmicutes; c__Bacilli; o__Bacillales; f__Bacillaceae; g__Bacillus; s__coagulans
OTU_2347	k__Bacteria; p__Firmicutes; c__Bacilli; o__Lactobacillales; f__Lactobacillaceae; g__Pediococcus; s__
OTU_1638	k__Bacteria; p__Proteobacteria; c__Alphaproteobacteria; o__Rhizobiales; f__Phyllobacteriaceae; g__; s__
OTU_153	k__Bacteria; p__Firmicutes; c__Clostridia; o__Clostridiales; f__Lachnospiraceae; g__Coprococcus; s__
OTU_150	k__Bacteria; p__Actinobacteria; c__Actinobacteria; o__Actinomycetales; f__Actinopolysporaceae; g__; s__
OTU_151	k__Bacteria; p__Firmicutes; c__Clostridia; o__Clostridiales; f__Lachnospiraceae; g__; s__
OTU_18	k__Bacteria; p__Proteobacteria; c__Gammaproteobacteria; o__Enterobacteriales; f__Enterobacteriaceae; g__Enterobacter; s__
OTU_19	k__Bacteria; p__Firmicutes; c__Bacilli; o__Bacillales; f__Bacillaceae; g__Bacillus; s_2e-141
OTU_14	k__Bacteria; p__Proteobacteria; c__Alphaproteobacteria; o__Rhodobacterales; f__Rhodobacteraceae; g__; s__
OTU_15	k__Bacteria; p__Actinobacteria; c__Actinobacteria; o__Actinomycetales; f__Streptomycetaceae; g__Streptomyces; s__
OTU_16	k__Bacteria; p__Proteobacteria; c__Gammaproteobacteria; o__Enterobacteriales; f__Enterobacteriaceae; g__Enterobacter; s__gergoviae
OTU_17	k__Bacteria; p__Firmicutes; c__Bacilli; o__Lactobacillales; f__Lactobacillaceae; g__; s__
OTU_10	k__Bacteria; p__Actinobacteria; c__Actinobacteria; o__Actinomycetales; f__Micrococcaceae; g__; s__
OTU_11	k__Bacteria; p__Actinobacteria; c__Actinobacteria; o__Actinomycetales; f__Dermabacteraceae; g__Brachybacterium; s__
OTU_12	k__Bacteria; p__Actinobacteria; c__Actinobacteria; o__Actinomycetales; f__Brevibacteriaceae; g__Brevibacterium; s__aureum
OTU_13	k__Bacteria; p__Bacteroidetes; c__Sphingobacteriia; o__Sphingobacteriales; f__Sphingobacteriaceae; g__Sphingobacterium; s__
OTU_2165	k__Bacteria; p__Actinobacteria; c__Actinobacteria; o__Actinomycetales; f__Microbacteriaceae; g__Leucobacter; s__
OTU_154	k__Bacteria; p__Firmicutes; c__Bacilli; o__Bacillales; f__Paenibacillaceae; g__Paenibacillus; s__
OTU_2043	k__Bacteria; p__Actinobacteria; c__Actinobacteria; o__Actinomycetales; f__Microbacteriaceae; g__Pseudoclavibacter; s__bifida
OTU_675	k__Bacteria; p__Actinobacteria; c__Actinobacteria; o__Actinomycetales; f__Micrococcaceae; g__; s__
OTU_330	k__Bacteria; p__Firmicutes; c__Bacilli; o__Bacillales; f__Paenibacillaceae; g__Paenibacillus; s__
OTU_83	k__Bacteria; p__Actinobacteria; c__Actinobacteria; o__Actinomycetales; f__Promicromonosporaceae; g__Xylanimicrobium; s__pachnodae
OTU_82	k__Bacteria; p__Firmicutes; c__Bacilli; o__Bacillales; f__Bacillaceae; g__Virgibacillus; s__halophilus
OTU_80	k__Bacteria; p__Bacteroidetes; c__Sphingobacteriia; o__Sphingobacteriales; f__Sphingobacteriaceae; g__; s__
OTU_222	k__Bacteria; p__Proteobacteria; c__Betaproteobacteria; o__Burkholderiales; f__Alcaligenaceae; g__; s__
OTU_84	k__Bacteria; p__Bacteroidetes; c__Sphingobacteriia; o__Sphingobacteriales; f__Sphingobacteriaceae; g__Olivibacter; s__
OTU_179	k__Bacteria; p__Bacteroidetes; c__Sphingobacteriia; o__Sphingobacteriales; f__Sphingobacteriaceae; g__; s__
OTU_89	k__Bacteria; p__Actinobacteria; c__Actinobacteria; o__Actinomycetales; f__Nocardiaceae; g__Rhodococcus; s__ruber
OTU_1905	k__Bacteria; p__Firmicutes; c__Bacilli; o__Bacillales; f__Bacillaceae; g__Bacillus; s__coagulans

OTU_254	k__Bacteria; p__Proteobacteria; c__Alphaproteobacteria; o__Caulobacterales; f__Caulobacteraceae; g__Brevundimonas; s__diminuta
OTU_701	k__Bacteria; p__Firmicutes; c__Bacilli; o__Bacillales; f__Paenibacillaceae; g__Paenibacillus; s__
OTU_2652	k__Bacteria; p__Actinobacteria; c__Actinobacteria; o__Actinomycetales; f__Dermabacteraceae; g__Brachybacterium; s__
OTU_2172	k__Bacteria; p__Actinobacteria; c__Actinobacteria; o__Actinomycetales; f__Micrococcaceae; g__; s__
OTU_2517	k__Bacteria; p__Actinobacteria; c__Actinobacteria; o__Actinomycetales; f__Pseudonocardiaceae; g__Saccharopolyspora; s__
OTU_1644	k__Bacteria; p__Firmicutes; c__Bacilli; o__Bacillales; f__Paenibacillaceae; g__Paenibacillus; s__
OTU_1976	k__Bacteria; p__Firmicutes; c__Bacilli; o__Bacillales; f__Bacillaceae; g__Anaerobacillus; s__
OTU_1709	k__Bacteria; p__Firmicutes; c__Bacilli; o__Bacillales; f__Staphylococcaceae; g__Staphylococcus; s__succinus
OTU_1022	k__Bacteria; p__Proteobacteria; c__Betaproteobacteria; o__Burkholderiales; f__Alcaligenaceae; g__; s__
OTU_2599	k__Bacteria; p__Actinobacteria; c__Actinobacteria; o__Actinomycetales; f__Brevibacteriaceae; g__Brevibacterium; s__
OTU_1508	k__Bacteria; p__Firmicutes; c__Bacilli; o__Bacillales; f__Paenibacillaceae; g__Paenibacillus; s__
OTU_1558	k__Bacteria; p__Firmicutes; c__Bacilli; o__Bacillales; f__Staphylococcaceae; g__Staphylococcus; s__succinus
OTU_2329	k__Bacteria; p__Proteobacteria; c__Alphaproteobacteria; o__Rhodobacterales; f__Rhodobacteraceae; g__Paracoccus; s__
OTU_2202	k__Bacteria; p__Actinobacteria; c__Actinobacteria; o__Actinomycetales; f__Brevibacteriaceae; g__Brevibacterium; s__
OTU_1934	k__Bacteria; p__Firmicutes; c__Bacilli; o__Bacillales; f__Bacillaceae; g__Bacillus; s__coagulans
OTU_2344	k__Bacteria; p__Actinobacteria; c__Actinobacteria; o__Actinomycetales; f__Streptomycetaceae; g__Streptomyces; s__
OTU_2456	k__Bacteria; p__Actinobacteria; c__Actinobacteria; o__Actinomycetales; f__Beutenbergiaceae; g__Serinibacter; s__salmoneus
OTU_1807	k__Bacteria; p__Actinobacteria; c__Actinobacteria; o__Actinomycetales; f__Rarobacteraceae; g__Rarobacter; s__
OTU_2511	k__Bacteria; p__Actinobacteria; c__Actinobacteria; o__Actinomycetales; f__Microbacteriaceae; g__Leucobacter; s__
OTU_109	k__Bacteria; p__Proteobacteria; c__Betaproteobacteria; o__Burkholderiales; f__Burkholderiaceae; g__Pandoraea; s__
OTU_348	k__Bacteria; p__Bacteroidetes; c__Flavobacteriia; o__Flavobacteriales; f__Flavobacteriaceae; g__; s__
OTU_665	k__Bacteria; p__Actinobacteria; c__Actinobacteria; o__Actinomycetales; f__Nocardioidaceae; g__Aeromicrobium; s__
OTU_667	k__Bacteria; p__Firmicutes; c__Bacilli; o__Bacillales; f__Bacillaceae; g__Bacillus; s_1e-136
OTU_1504	k__Bacteria; p__Proteobacteria; c__Alphaproteobacteria; o__Rhizobiales; f__Brucellaceae; g__; s__
OTU_144	k__Bacteria; p__Firmicutes; c__Bacilli; o__Bacillales; f__Paenibacillaceae; g__Paenibacillus; s__
OTU_108	k__Bacteria; p__Firmicutes; c__Bacilli; o__Lactobacillales; f__Leuconostocaceae; g__; s__
OTU_652	k__Bacteria; p__Firmicutes; c__Bacilli; o__Lactobacillales; f__Aerococcaceae; g__Facklamia; s__
OTU_141	k__Bacteria; p__Firmicutes; c__Bacilli; o__Bacillales; f__Paenibacillaceae; g__; s__
OTU_77	k__Bacteria; p__Bacteroidetes; c__Flavobacteriia; o__Flavobacteriales; f__[Weeksellaceae]; g__; s__
OTU_147	k__Bacteria; p__Proteobacteria; c__Betaproteobacteria; o__Burkholderiales; f__Burkholderiaceae; g__Burkholderia; s__
OTU_72	k__Bacteria; p__Firmicutes; c__Bacilli; o__Lactobacillales; f__Lactobacillaceae; g__Pediococcus; s__
OTU_73	k__Bacteria; p__Bacteroidetes; c__Sphingobacteriia; o__Sphingobacteriales; f__Sphingobacteriaceae; g__; s__
Bacteria Raw+Ripened Pu-erh	
OTU_2616	k__Bacteria; p__Actinobacteria; c__Actinobacteria; o__Actinomycetales; f__Pseudonocardiaceae; g__Saccharopolyspora; s__
OTU_1665	k__Bacteria; p__Proteobacteria; c__Gammaproteobacteria; o__Pseudomonadales; f__Pseudomonadaceae; g__Pseudomonas; s__
OTU_90	k__Bacteria; p__Proteobacteria; c__Gammaproteobacteria; o__Pseudomonadales; f__Pseudomonadaceae; g__Pseudomonas; s__
OTU_99	k__Bacteria; p__Proteobacteria; c__Gammaproteobacteria; o__Xanthomonadales; f__Xanthomonadaceae; g__; s__
OTU_2480	k__Bacteria; p__Firmicutes; c__Bacilli; o__Bacillales; f__Staphylococcaceae; g__Staphylococcus; s__succinus
OTU_1075	k__Bacteria; p__Proteobacteria; c__Betaproteobacteria; o__Burkholderiales; f__Comamonadaceae; g__Polaromonas; s__
OTU_4	k__Bacteria; p__Actinobacteria; c__Actinobacteria; o__Actinomycetales; f__Pseudonocardiaceae; g__Saccharopolyspora; s__
OTU_134	k__Bacteria; p__Firmicutes; c__Bacilli; o__Bacillales; f__Alicyclobacillaceae; g__Alicyclobacillus; s__
OTU_24	k__Bacteria; p__Proteobacteria; c__Gammaproteobacteria; o__Pseudomonadales; f__Moraxellaceae; g__Acinetobacter; s__
OTU_670	k__Bacteria; p__Firmicutes; c__Bacilli; o__Bacillales; f__Bacillaceae; g__Bacillus; s_2e-141
OTU_2391	k__Bacteria; p__Firmicutes; c__Bacilli; o__Bacillales; f__Planococcaceae; g__Planococcus; s__maitriensis
OTU_1769	k__Bacteria; p__Firmicutes; c__Bacilli; o__Bacillales; f__Planococcaceae; g__Planomicrobium; s__

Table C. ANOSIM and ADONIS test of four variables on fungal/bacterial community in raw/ripened Pu-erh

			Fungi		Bacteria		
			Binary-Jaccard	Bray-Curtis	Binary-Jaccard	Bray-Curtis	weighted-Unifrac
Age stage on raw Pu-erh	ANOSIM	R	0.139	0.184	0.180	0.326	0.344
		P	0.103	0.069	0.098	0.041	0.040
	ADONIS	R^2	0.195	0.201	0.206	0.337	0.463
		P	0.006	0.058	0.090	0.023	0.019
Age stage on ripened Pu-erh	ANOSIM	R	-0.023	-0.020	0.074	0.261	0.057
		P	0.514	0.492	0.209	0.022	0.245
	ADONIS	R^2	0.088	0.063	0.077	0.132	0.111
		P	0.090	0.390	0.234	0.016	0.122
Producer on raw Pu-erh	ANOSIM	R	0.093	0.112	0.043	0.169	0.228
		P	0.271	0.201	0.372	0.142	0.123
	ADONIS	R^2	0.317	0.331	0.108	0.321	0.446
		P	0.119	0.179	0.100	0.239	0.101
Producer on ripened Pu-erh [a]	ANOSIM	R	0.178 (0.148)	-0.027	0.181 (0.370)	0.064	-0.004
		P	0.009 (0.305)	0.521	0.045 (0.204)	0.244	0.441
	ADONIS	R^2	0.264 (0.219)	0.188	0.265 (0.261)	0.229	0.186
		P	0.009 (0.491)	0.486	0.017 (0.210)	0.206	0.506
Plant status on ripened Pu-erh	ANOSIM	R	-0.022	0.196	0.084	0.170	0.127
		P	0.548	0.036	0.148	0.045	0.080
	ADONIS	R^2	0.075	0.167	0.071	0.096	0.086
		P	0.288	0.054	0.353	0.137	0.217
Tea form on raw Pu-erh	ANOSIM	R	0.204	-0.098	0.009	-0.146	-0.140
		P	0.037	0.856	0.428	0.888	0.927
	ADONIS	R^2	0.085	0.053	0.092	0.048	0.031
		P	0.134	0.739	0.425	0.925	0.877
Tea form on ripened Pu-erh	ANOSIM	R	0.194	-0.112	0.227	0.129	-0.060
		P	0.094	0.823	0.061	0.178	0.619
	ADONIS	R^2	0.087	0.023	0.107	0.090	0.052
		P	0.110	0.870	0.020	0.159	0.580

[a] When we tested for the effect of producers on fungal/bacteria community in ripened Pu-erh tea, significant *P* values (outside parentheses) were initially obtained using the Binary-Jaccard distance matrices. But when we focused on just two producers whose samples have similar age range, the *P* values (within parentheses) were not significant again.

Table D. Mantel test between the fungal and bacterial communities based on either Binary-Jaccard or Bray-Curtis distance matrices

Tea type	No. of samples	Binary-Jaccard		Bray-Curtis	
		r	***P*-value**	***r***	***P*-value**
Raw Pu-erh	12	-0.031	0.866	-0.018	0.896
Ripened Pu-erh	16	0.200	0.216	0.410	0.004
	9	0.142	0.493	0.063	0.730

* For raw Pu-erh, only the 12 samples with sufficient number of sequences were tested. For ripened Pu-erh, we tested both all samples and just the nine samples with pure *Camellia sinensis* detection.

Table E. The first 15 most abundant bacterial OTUs in fresh tea leaf, raw Pu-erh, and ripened Pu-erh

Abundance Rank	Fresh tea leaf			Raw Pu-erh			Ripened Pu-erh		
	**OTU ID**	**Bacterial taxon**	**Occurrence**	**OTU ID**	**Bacterial taxon**	**Occurrence**	**OTU ID**	**Bacterial taxon**	**Occurrence**
1	OTU_21	Burkholderiales sp.	4	OTU_2616	*Saccharopolyspora sp.*	14	OTU_2480	*Staphylococcus succinus*	16
2	OTU_65	*Methylobacterium* sp.	7	OTU_25	*Streptomyces sp.*	3	OTU_1748	Micrococcaceae sp.	16
3	OTU_959	Phormidiaceae sp.	6	OTU_4	*Saccharopolyspora sp.*	8	OTU_6	*Bacillus coagulans*	16
4	OTU_319	*Pseudomonas viridiflava*	5	OTU_2557	Enterobacteriaceae sp.	13	OTU_2648	*Brevibacterium aureum*	16
5	OTU_756	*Curtobacterium* sp.	6	OTU_61	*Turicibacter sp.*	3	OTU_2123	*Brachybacterium sp.*	16
6	OTU_64	*Sphingomonas yabuuchiae*	6	OTU_1224	Enterobacteriaceae sp.	5	OTU_13	*Sphingobacterium sp.*	15
7	OTU_140	*Luteibacter rhizovicinus*	4	OTU_24	*Acinetobacter sp.*	15	OTU_17	Lactobacillaceae sp.	16
8	OTU_2374	Burkholderiales sp.	1	OTU_2480	*Staphylococcus succinus*	15	OTU_16	*Enterobacter gergoviae*	16
9	OTU_186	*Oceanobacillus sp.*	1	OTU_75	*Sciscionella marina*	4	OTU_19	*Bacillus sp.*	16
10	OTU_1331	*Sphingomonas sp.*	6	OTU_79	Peptostreptococcaceae sp.	13	OTU_2616	*Saccharopolyspora sp.*	15
11	OTU_2480	*Staphylococcus succinus*	5	OTU_44	Oxalobacteraceae sp.	14	OTU_18	*Enterobacter sp.*	16
12	OTU_145	Microbacteriaceae sp.	6	OTU_81	*Amycolatopsis sp.*	1	OTU_15	*Streptomyces sp.*	12
13	OTU_2651	*Pedobacter sp.*	1	OTU_87	*Prevotella sp.*	1	OTU_20	*Listeria grayi*	16
14	OTU_125	*Methylobacterium adhaesivum*	6	OTU_118	Clostridiales sp.	12	OTU_1569	*Bacillus thermoamylovorans*	16
15	OTU_383	*Paenibacillus sp.*	1	OTU_86	Clostridiaceae sp.	12	OTU_1799	*Brevibacterium aureum*	16

Occurrence indicates the detection of a given OTU in the seven fresh leaf samples, the 15 raw Pu-erh samples, or the 16 ripened Pu-erh samples.
